# Extremely low nucleotide diversity among thirty-six new chloroplast genome sequences from *Aldama* (Heliantheae, Asteraceae) and comparative chloroplast genomics analyses with closely related genera

**DOI:** 10.7717/peerj.10886

**Published:** 2021-02-24

**Authors:** Benoit Loeuille, Verônica Thode, Carolina Siniscalchi, Sonia Andrade, Magdalena Rossi, José Rubens Pirani

**Affiliations:** 1 Departamento de Botânica, Universidade Federal de Pernambuco, Recife, Pernambuco, Brazil; 2 Instituto de Biociências, Universidade Federal do Rio Grande do Sul, Porto Alegre, Rio Grande do Sul, Brazil; 3 Department of Biological Sciences, Mississippi State University, Mississippi State, MS, United States of America; 4 Departamento de Genética e Biologia Evolutiva, Universidade de São Paulo, São Paulo, São Paulo, Brazil; 5 Departamento de Botânica, Universidade de São Paulo, São Paulo, São Paulo, Brazil

**Keywords:** Complete plastome, Compositae, ycf2 gene, rbcL positive selection, Species-level plastome evolution

## Abstract

*Aldama* (Heliantheae, Asteraceae) is a diverse genus in the sunflower family. To date, nearly 200 Asteraceae chloroplast genomes have been sequenced, but the plastomes of *Aldama* remain undescribed. Plastomes in Asteraceae usually show little sequence divergence, consequently, our hypothesis is that species of *Aldama* will be overall conserved. In this study, we newly sequenced 36 plastomes of *Aldama* and of five species belonging to other Heliantheae genera selected as outgroups (i.e., *Dimerostemma asperatum, Helianthus tuberosus, Iostephane heterophylla, Pappobolus lanatus* var*. lanatus, and Tithonia diversifolia)*. We analyzed the structure and gene content of the assembled plastomes and performed comparative analyses within *Aldama* and with other closely related genera. As expected, *Aldama* plastomes are very conserved, with the overall gene content and orientation being similar in all studied species. The length of the plastome is also consistent and the junction between regions usually contain the same genes and have similar lengths. A large ∼20 kb and a small ∼3 kb inversion were detected in the Large Single Copy (LSC) regions of all assembled plastomes, similarly to other Asteraceae species. The nucleotide diversity is very low, with only 1,509 variable sites in 127,466 bp (i.e., 1.18% of the sites in the alignment of 36 *Aldama* plastomes, with one of the IRs removed, is variable). Only one gene, *rbcL*, shows signatures of positive selection. The plastomes of the selected outgroups feature a similar gene content and structure compared to *Aldama* and also present the two inversions in the LSC region. Deletions of different lengths were observed in the gene *ycf2*. Multiple SSRs were identified for the sequenced *Aldama* and outgroups. The phylogenetic analysis shows that *Aldama* is not monophyletic due to the position of the Mexican species *A. dentata*. All Brazilian species form a strongly supported clade. Our results bring new understandings into the evolution and diversity of plastomes at the species level.

## Introduction

In the last two decades, the rise of next generation sequencing (NGS) methods have dramatically changed the field of biology. The large amount of affordable genomic data produced by NGS allows addressing biodiversity questions at a genome-wide scale (*Parks, Cronn & Liston, 2009; Koboldt et al., 2013; Goodwin, McPherson & McCombie, 2016*), with an unprecedented level of detail (*Porter & Hajibabei, 2018; Krehenwinkel, Pomerantz & Prost, 2019*). NGS methods have been applied from conservation genetics (*Hunter et al., 2018; Breed et al., 2019*) to the sequencing of ancient genomes (*Gutaker & Burbano, 2017; Estrada et al., 2018*). In plant systematics, NGS offers opportunities to investigate phylogenomic and phylogeographic processes with unparalleled precision and depth (*Straub et al., 2012; Hörandl & Appelhans, 2015*): either at a macroevolutionary level, mostly through inference of robust, dated phylogenies to investigate species diversification and other large-scale biodiversity patterns (e.g., Léveillé-Bourret et al., 2018; Pouchon et al., 2018; Wong et al., 2020), or at a microevolutionary level to study population dynamics (McCormack et al., 2013; Morris & Shaw, 2018).

Chloroplast genome size varies from 120 to 170 kb across angiosperms, coding for 110–130 genes (∼80 proteins, ∼30 tRNAs and four rRNAs). Chloroplast organization is especially maintained throughout land plant evolution, with two copies of an inverted repeat (IR) region that separate two regions of single-copy genes: the large and small single copy regions (LSC and SSC, respectively). Despite the conserved gene content and organization shared among plastomes in higher plant lineages, some structural changes can occur (e.g., gene and intron losses, inversions, deletions, and duplications), as well as point mutations, especially in the non-protein coding regions that account for ∼50% of the genome (*Raubeson & Jansen, 2005; Ruhlman & Jansen, 2014*). Therefore, this level of variability makes chloroplast sequences suitable for phylogenetic analyses (*Gitzendanner et al., 2018*). They have been used as phylogenetic markers due to a series of advantages: small genome size, high copy number per cell, conserved gene order, uniparental inheritance (usually maternal, rarely biparental) and lack of recombination (*Raubeson & Jansen, 2005*).

The inference of evolutionary relationships among land plants has relied mainly on chloroplast sequences at diverse taxonomic levels (*Freitas et al., 2018*; *Gitzendanner et al., 2018*). NGS has greatly simplified the acquisition of whole chloroplast genome sequences (*Parks, Cronn & Liston, 2009; Stull et al., 2013*), with more than 4,500 plastomes sequenced to date (Genbank: https://www.ncbi.nlm.nih.gov/refseq/, accessed on July 2020), and recent publication of numerous plastome phylogenies (e.g., Sun et al., 2016; Givnish et al., 2018; Kuo et al., 2018; Saarela et al., 2018; Li et al., 2019). Structural changes in the chloroplast genome can also serve as powerful phylogenetic evidence, with one of the most notable examples found in the family Asteraceae. Jansen & Palmer (1987a) and Jansen & Palmer (1987b), through comparative restriction site and gene mapping studies, found a 22.8 kb inversion in the LSC region shared by all studied members of the family, except for the early-diverging lineage Barnadesieae, which was later confirmed as the sister-group to the rest of Asteraceae (*Kim, Choi & Jansen, 2005; Funk et al., 2009; Mandel et al., 2019*).

Asteraceae is one of the largest families of flowering plants, with 25,000–35,000 species, occurring in all continents and in nearly all types of vegetations (Mandel et al., 2019). This high species diversity is presumably linked to a series of whole genome duplications and paleopolyploidization events (*Barker et al., 2008; Barker et al., 2016; Huang et al., 2016*). One of these events was previously identified at the stem leading to the Heliantheae Alliance clade (*Huang et al., 2016*), being shortly followed by an acceleration in diversification in the phytomelanic fruit clade (i.e., all Heliantheae Alliance except the tribe Helenieae), which resulted in ∼457 genera and ∼5,500 species (*Funk et al., 2009; Panero & Crozier, 2016; Mandel et al., 2019*).

*Aldama* is a genus of the Heliantheae Alliance (Heliantheae: Helianthinae) with ∼120 species (*Schilling & Panero, 2011; Magenta & Pirani, 2014*), occurring in the southwestern USA and in most of South America, in mountainous regions and open vegetations. They are perennial herbs, with heads containing neutral ray florets, pappus composed by two awns and several short, deciduous or persistent squamellae (*Magenta, Loeuille & Pirani, 2017*). Previously to the phylogenetic analysis by Schilling & Panero (2011), the polyphyletic genus *Viguiera* contained most of the species currently placed in *Aldama*, including the 36 Brazilian species that are now placed in the latter. However, this new circumscription remains uncertain due to the small sampling of South American taxa in Schilling & Panero’s (2011) study and the absence of an unambiguous morphological synapomorphy to define *Aldama*. The marked incongruences among subtribal level phylogenies, either based on nuclear regions (*Schilling & Panero, 2011*) or those based on a chloroplast restriction site dataset (*Schilling & Jansen, 1989; Schilling & Panero, 1996a*), indicate that introgression has played a significant role in the evolutionary history of this group (*Schilling & Panero, 1996b*). Nonetheless, the low level of DNA sequence divergence, either in ribosomal or chloroplast data (*Schilling et al., 2000; Schilling & Panero, 2011*), has been an obstacle to a clear understanding of evolutionary relationships in Helianthinae.

The chloroplast genome of Asteraceae, except for the Barnadesieae lineage, is characterized by two inversions: the previously mentioned 22.8 kb inversion in the LSC region and a second, 3.3 kb inversion, nested within the larger one (*Kim, Choi & Jansen, 2005; Timme et al., 2007*). The plastome size in the family varies from 149,5 to 153,7 kb (*Choi & Park, 2015; Lu et al., 2016*) and the gene content is relatively conserved: from 111 to 115 different genes, including 79 to 83 protein-coding genes, four rRNAs genes, and 29–30 distinct tRNAs (*Wang et al., 2015; Salih et al., 2017; Lin et al., 2019; Wang et al., 2020*). Some variations have been found in the gene structure and tRNA abundance, and in some regions the nucleotide diversity is higher than 5% (*Wang et al., 2015*). These more diverse regions can be used as phylogenetic markers and even to identify cryptic lineages in some cases (*Wang et al., 2015; Salih et al., 2017*). Several plastome phylogenies have been published recently (*Pouchon et al., 2018; Cho et al., 2019; Zhang et al., 2019; Knope et al., 2020*), two of them involving lineages of the Heliantheae Alliance (the *Espeletia* complex, Pouchon et al., 2018, and the genus *Bidens* Knope et al., 2020). These recent studies also reveal incongruence between plastid and nuclear phylogenies, likely due to the exchange of plastids between geographically close species, regardless of their morphological similarities or nuclear phylogenetic distance (*Pouchon et al., 2018*), or to hybridization events and/or incomplete lineage sorting (*Knope et al., 2020*).

With the aim to increase our comprehension of chloroplast genome characteristics, structural diversity, and evolution at low taxonomic levels in the tribe Heliantheae, we sequenced the plastome of 33 species of *Aldama* (Heliantheae, Asteraceae) representing a wide range of the morphological diversity in the genus. Additionally, the plastome of five species belonging to other Heliantheae genera, selected as outgroups, were also sequenced (i.e., *Dimerostemma asperatum* S.F. Blake, *Helianthus tuberosus* L., *Iostephane heterophylla* (Cav.) Benth., *Pappobolus lanatus* (Heiser) Panero var. *lanatus*, and *Tithonia diversifolia* (Hemsl.) A. Gray). We analyzed the structure and content of the assembled plastomes, performed comparative analyses within *Aldama* and with other closely related genera, registered selection patterns within *Aldama* chloroplast genes, identified putative repeated regions, and reconstructed phylogenetic relationships.

## Materials & Methods

### Sampling, DNA preparation, and sequencing

We sampled 36 individuals of *Aldama*, representing 33 species, and one individual each of five species belonging to other Heliantheae genera, four of them from subtribe Helianthinae (*Helianthus tuberosus*, *Iostephane heterophylla*, *Pappobolus lanatus* var. *lanatus*, and *Tithonia diversifolia*) and one from subtribe Ecliptinae (*Dimerostemma asperatum*), which were selected as outgroups based on other studies (*Panero, 2007; Schilling & Panero, 2011*) (Table 1, Data S1). Total genomic DNA was extracted from silica-gel-dried leaves collected in the field using the commercial kit E.Z.N.A.^®^ SQ Plant DNA Kit (Omega Bio-Tek Inc., Norcross, GA, USA) following the manufacturer’s instructions except by the addition of ascorbic acid and polyvinylpyrrolidone in the SQ1 buffer. The integrity of genomic DNA was assessed by performing electrophoresis in a 1% agarose gel and DNA concentrations were measured with spectrophotometric analysis with Epoch Micro-Volume Spectrophotometer System (BioTek). DNA of each sample was diluted to a final volume of 20 µL at 2.5 ng/µL, quantified with Qubit fluorometer (Invitrogen) and used to construct Illumina Nextera libraries (Illumina, San Diego, CA, USA) following the manufacturer’s instructions. Plastome capture was carried out with a customized SureSelectXT Custom 1kb-499kb library^®^ (Agilent Technologies) target enrichment panel, using biotinylated RNA baits developed based on the *H. annuus* plastome sequence (Data S2). This customized panel includes both the complete plastome and several low copy nuclear loci (unpublished data), thus ensuring high sequencing coverage. DNA library preparation, target enrichment and sequencing were carried out at Laboratório Multiusuários Centralizado (ESALQ-USP, Piracicaba - SP, Brazil). Sequencing was conducted using an Illumina HiSeq platform in paired-end mode, with 300 bp read lenght.

**Table 1:** Taxa, voucher, reference, GenBank accession numbers and summary of the plastomes sequenced in this study.

**Species**	**Origin/Voucher/ Herbarium**	**GenBank (accession)**	**Plastome length (bp)**	**LSC length (bp)**	**IR length (bp)**	**SSC length (bp)**	**Inv1**	**Inv2**
*Aldama anchusifolia* (DC.) E.E.Schill. & Panero	Brazil/Filartiga 8/ESA	MN337902	151,330	83,717	24,633	18,347	22,360	3,298
*A. arenaria* 1 (Baker) E.E.Schill. & Panero	Brazil/Magenta 275/SPF	MN337903	151,346	83,732	24,633	18,349	22,386	3,319
*A. arenaria* 2 (Baker) E.E.Schill. & Panero	Brazil/Magenta 383/SPF	MN337904	151,345	83,735	24,632	18,346	22,367	3,309
*A. aspilioides* (Baker) E.E.Schill. & Panero	Brazil/Filartiga 18/ESA	MN337905	151,362	83,742	24,633	18,359	22,380	3,313
*A. bakeriana* (S.F.Blake) E.E.Schill. & Panero	Brazil/Loeuille 867/SPF	MN337906	151,376	83,757	24,631	18,345	22,396	3,328
*A. bracteata* (Gardner) E.E.Schill. & Panero	Brazil/Filartiga 15/ESA	MN337907	151,339	83,714	24,632	18,361	22,378	3,312
*A. canescens* (B.L. Rob.) E.E.Schill. & Panero	Mexico/Schilling 17/TENN	MN337908	151,386	83,719	24,666	18,346	22,380	3,311
*A. corumbensis* (Malme) Magenta & Pirani	Brazil/Loeuille 909/SPF	MN337909	151,327	83,720	24,632	18,342	22,372	3,309
*A. dentata* 1 La Llave	Mexico/Schilling 331/TENN	MN337910	151,303	83,635	24,665	18,353	22,350	3,308
*A. dentata* 2 La Llave	Mexico/Schilling 333/TENN	MN356024	151,336	83,680	24,664	18,345	22,355	3,302
*A. discolor* (Baker) E.E.Schill. & Panero	Brazil/Bombo 72/ESA	MN356025	151,360	83,766	24,632	18,346	22,380	3,315
*A. excelsa* (Willd.) E.E.Schill. & Panero	Mexico/Schilling H2481/TENN	MN356026	151,312	83,684	24,650	18,325	22,376	3,313
*A. filifolia* (Sch.Bip. ex Baker) E.E.Schill. & Panero	Brazil/Loeuille 849/SPF	MN337890	151,333	83,711	24,633	18,347	22,370	3,309
*A. fusiformis* (S.F.Blake) E.E.Schill. & Panero	Peru/Siniscalchi 398/SPF	MN337891	151,314	83,728	24,620	18,344	22,376	3,309
*A. gardneri* (Baker) E.E.Schill. & Panero	Brazil/Filartiga 16/ESA	MN337892	151,312	83,694	24,633	18,343	22,362	3,308
*A. goyazii* E.E.Schill. & Panero	Brazil/Magenta 716/SPF	MN337893	151,279	83,687	24,632	18,326	22,366	3,308
*A. grandiflora* (Gardner) E.E.Schill. & Panero	Brazil/Loeuille 750/SPF	MN337894	151,353	83,761	24,633	18,326	22,368	3,309
*A. kunthiana* (Gardner) E.E.Schill. & Panero	Brazil/Silva s.n./ESA 122873	MN337895	151,327	83,699	24,633	18,360	22,378	3,315
*A. linearis* (Cav.) E.E.Schill. & Panero	Mexico/Schilling 70/TENN	MN337896	151,314	83,665	24,667	18,334	22,379	3,307
*A. macrorhiza* (Baker) E.E.Schill. & Panero	Brazil/Magenta 476/SPF	MN337897	151,314	83,701	24,633	18,347	22,372	3,309
*A. megapotamica* (Malme) Magenta & Pirani	Brazil/Magenta 502/SPF	MN337898	151,363	83,731	24,642	18,346	22,389	3,327
*A. nudibasilaris* (S.F. Blake) E.E.Schill. & Panero	Brazil/Filartiga 1/ESA	MN337899	151,316	83,704	24,633	18,344	22,375	3,310
*A. nudicaulis* (Baker) E.E.Schill. & Panero	Brazil/Loeuille 734/SPF	MN337900	151,333	83,731	24,633	18,334	22,376	3,312
*A. pilosa* (Baker) E.E.Schill. & Panero	Brazil/Filartiga 10/ESA	MN337901	151,354	83,746	24,633	18,342	22,372	3,309
*A. revoluta* (Meyen) E.E.Schill. & Panero	Chile/Loeuille 799/SPF	MN356027	151,376	83,742	24,638	18,353	22,375	3,303
*A. robusta* (Gardner) E.E.Schill. & Panero	Brazil/Bringel 985/UB	MN356028	151,343	83,734	24,633	18,344	22,373	3,309
*A. rubra* (Magenta & Pirani) E.E.Schill. & Panero	Brazil/Magenta 388/SPF	MN356029	151,322	83,708	24,634	18,344	22,375	3,309
*A. santacatarinensis* (H.Rob. & A.J.Moore) E.E.Schill. & Panero	Brazil/Magenta 706/SPF	MN356030	151,362	83,738	24,635	18,345	22,368	3,307
*A. squalida* (S.Moore) E.E.Schill. & Panero	Brazil/Loeuille 790/SPF	MN356031	151,399	83,782	24,631	18,346	22,384	3,317
*A. tenuifolia* (Gardner) E.E.Schill. & Panero	Brazil/Silva s.n./ESA 122870	MN356032	151,372	83,759	24,634	18,344	22,380	3,314
*A. trichophylla* 1 (Dusén) Magenta	Brazil/Magenta 390/SPF	MN337911	151,353	83,749	24,633	18,338	22,371	3,308
*A. trichophylla* 2 (Dusén) Magenta	Brazil/Magenta 561/SPF	MN311247	151,301	83,690	24,632	18,347	22,369	3,308
*A. tuberosa* (Griseb.) E.E.Schill. & Panero	Brazil/Loeuille 719/SPF	MN356033	151,323	83,715	24,634	18,338	22,376	3,309
*A. tucumanensis* (Hook. & Arn.) E.E.Schill. & Panero	Argentina/Heiden 1837/SPF	MN356034	151,336	83,724	24,634	18,342	22,375	3,314
*A. veredensis* (Magenta & Pirani) E.E.Schill. & Panero	Brazil/Loeuille 921/SPF	MN356035	151,331	83,715	24,632	18,350	22,373	3,308
*A. vernonioides* (Baker) E.E.Schill. & Panero	Brazil/Magenta 460/SPF	MN356036	151,221	83,676	24,607	18,329	22,366	3,309
**Outgroups**								
*Dimerostemma asperatum* S.F.Blake	Brazil/Siniscalchi 440/SPF	MT700540	151,862	84,107	24,704	18,347	22,472	3,295
*Helianthus tuberosus* L.	Chile/Loeuille 793/SPF	MT700541	151,242	83,634	24,632	18,344	22,411	3,312
*Iostephane heterophylla* (Cav.) Benth.	Mexico/Schilling 94/TENN	MT700542	151,495	83,812	24,640	18,317	22,396	3,305
*Pappobolus lanatus* var. *lanatus* (Heiser) Panero	Peru/Siniscalchi 386/SPF	MT700543	151,358	83,702	24,647	18,343	22,370	3,312
*Tithonia diversifolia* (Hemsl.) A.Gray	Brazil/Loeuille 678/SPF	MT700544	151,356	83,667	24,645	18,372	22,388	3,313

### Plastome assembly and annotation

Sequence quality was initially evaluated with FastQC 0.11.9 (*Andrews, 2010*) and trimmed for quality and contaminants with Trimmomatic (*Bolger, Lohse & Usadel, 2014*), using the SLIDINGWINDOW mode with a 5-base wide window and quality cut off of 20, dropping reads shorter than 36 bp. Due to the availability of a published chloroplast genome from a closely related taxon, *Helianthus. annuus* (GenBank accession NC_007977; *Timme et al., 2007)*, we opted for using reference assembly. This strategy also allows the assembly of off-target reads containing parts of the plastome sequence. The Bowtie2 plugin in Geneious (*Langmead & Salzberg, 2012*) was used to index the reference genome and to match the reads obtained from sequencing back to the reference genome. BAM files generated by Bowtie2 were used to calculate coverage of the sequencing with Samtools, using the “samtools depth” tool (Li et al., 2009), which gives the sequencing depth for each position in the final assembly. Basic statistics were used to summarize the coverage value for each taxon. Consensus sequences generated by the assembly were used for annotation and analysis.

Chloroplast genome annotations were carried out using Geneious 9.1.5 (*Kearse et al., 2012*), DOGMA (*Wyman, Jansen & Boore, 2004*), and BLAST (*Altschul, Gish & Miller, 1990; Altschul et al., 1997*). Open Reading Frames (ORFs) were confirmed by manually searching start and stop codons. The plastome of *Aldama trichophylla* was the first to be annotated, using *H. annuus* (GenBank accession NC_007977; Timme et al., 2007) as reference. This *Aldama* reference was subsequently used to annotate the remaining plastomes. The graphical illustration of the *A. trichophylla* annotated plastome was built in OGDRAW (*Lohse et al., 2013*). The junction sites between the LSC/IRa/SSC/IRb regions were manually determined in Geneious, with complete annotations for adjacent genes. The boundaries between the plastomes of one species representative of each genus sequenced in this study were plotted in IRscope (*Amiryousefi, Hyvönen & Poczai, 2018*; https://irscope.shinyapps.io/irapp/). All 41 newly-sequenced chloroplast genomes were submitted to GenBank (Table 1).

### Comparative analyses of chloroplast genomes

We performed comparative analyses among *Aldama* species and between *Aldama* and the five Heliantheae outgroup taxa. To avoid data duplication, one copy of the IRs of all chloroplast genomes was manually removed in all analyses.

Synteny and possible rearrangements were determined and identified through the comparison of the *A. trichophylla* plastome sequence with those from the five other Heliantheae genera. This analysis was completed in Mauve 2.4.0 (*Darling, Mau & Perna, 2010*, http://wolfe.gen.tcd.ie/GenomeVx), using progressiveMauve and MUSCLE 3.6 (*Edgar, 2004*) as alignment algorithm and internal aligner, respectively, with minimum locally collinear block (LCB) score and full alignment calculated automatically. We did not consider the plastomes to be collinear.

We aligned the 36 *Aldama* plastome sequences using the FFT-NS-2 method (*Katoh et al., 2002*) in MAFFT 7 (Katoh & Standley, 2013). This alignment was used to calculate the intrageneric nucleotide variability values (*π*) (*Nei & Li, 1979*). The *π* values were also calculated in a second dataset, using three *Aldama* species (*A. anchusifolia, A. dentata2, A. trichophylla*), the five genera from tribe Heliantheae sequenced in this study and three extra outgroups obtained from GenBank (*Echinacea paradoxa* (NC_034320), *H. annuus* (NC_007977), *H. argophyllus* (KU314500)). The sliding window analysis was performed in DnaSP 6.10 (*Rozas et al., 2017*) using a step size of 200 bp with window length of 800 bp. The *π* values were plotted using R (R Development Core Team, 2017). Percentage and total number of variable sites were estimated across *Aldama* plastomes using MEGA 7 (*Kumar, Stecher & Tamura, 2016*). 79 protein-coding genes were extracted from the 36 *Aldama* plastomes: i.e., *accD*, *atpA, B, E, F, H, I, ccsA, cemA, clpP, infA, matK, ndh A, B, C, D, E, F, G, H, I, J, K, petA, B, D, G, L, N, psaA, B, C, I, J, psbA, B, C, D, E, F, H, I, J, K, L, M, N, T, Z, rbcL, rpl2, 14, 16, 20, 22, 23, 32, 33, 36, rpoA, B, C1, C2, rps2, 3, 4, 7, 8, 11, 12, 14, 15, 16, 18, 19, ycf1, 2, 3,* and *4*. Each gene was separately aligned considering codon positions in Geneious, with the ClustalW plugin translation alignment tool (*Larkin et al., 2007*). MEGA 7 was used to estimate the number of variable sites within each of the 79 protein-coding genes. Based on the sequence alignment with 36 complete *Aldama* genomes, the ten most divergent plastomes were selected as representatives of the genus (i.e., *A. canescens*, *A. dentata* 2, *A. excelsa*, *A. filifolia*, *A. grandiflora*, *A. linearis*, *A. macrorhiza*, *A. trichophylla*, *A. rubra* and *A. veredensis*) and realigned in MAFFT as described above. We used mVISTA (*Frazer et al., 2004*) in Shuffle-LAGAN mode (*Brudno et al., 2003*) to identify variable regions within the genus by comparing these ten selected *Aldama* plastomes. To facilitate result visualization, in a second step, the procedure was repeated with three species of *Aldama* from different clades, selected according to the phylogeny inferred here (see results below; *A. anchusifolia*, *A. dentata* 2 and *A. trichophylla*), and the five other Heliantheae genera sequenced in this study. The annotated plastome of *A. trichophylla* was used as reference in both analyses.

### Selection on plastid genes

To investigate the presence positive selection signatures on the 79 plastid coding regions, Selecton 2.2 (*Stern et al., 2007*; http://selecton.tau.ac.il/index.html) was used to analyze the ratio of synonymous (Ks; silent) and non-synonymous (Ka; amino-acid altering) substitutions in the codon alignments for each region described above. We used the M8 (positive selection enabled; Yang et al., 2000) and M8a (null model; Swanson, Nielsen & Yang, 2003) models and likelihood scores were compared by a chi-square test with one degree of freedom. Results were considered significant when the probability was lower than 0.01.

### Simple sequence repeat analyses

Identification of microsatellites or Simple Sequence Repeats (SSRs; i.e.*,* tandem repeats of short, 1–6 bp-long DNA motifs) were performed using MISA (*Beier et al., 2017*) in the plastomes of the ten most divergent *Aldama* species (listed above) as representatives of the genus and of the five other Heliantheae genera sequenced in this study. Search for SSRs followed the following criteria: motif length between one and six nucleotides, with a minimum number of repetitions set as ten, five, and four units for mono-, di-, and trinucleotide SSRs, respectively, and three units each for tetra-, penta-, and hexanucleotide SSRs.

### Phylogenetic reconstruction

We reconstructed phylogenetic relationships among plastomes of 36 *Aldama* and using the five other Heliantheae species assembled here as outgroup. The FFT-NS-2 method (*Katoh et al., 2002*) in MAFFT 7 (Katoh & Standley, 2013) was used to align all plastomes with one of the IRs removed to avoid data duplication. The ML analysis was conducted in RAxML 8.2.9 (*Stamakis, 2014*) using the GTR+G model with node support assessed by fast-bootstrap (-f a) using 1,000 non-parametric bootstrap pseudo-replicates.

## Results

### Assembly and characteristics of the chloroplast genomes

**Figure 1:**
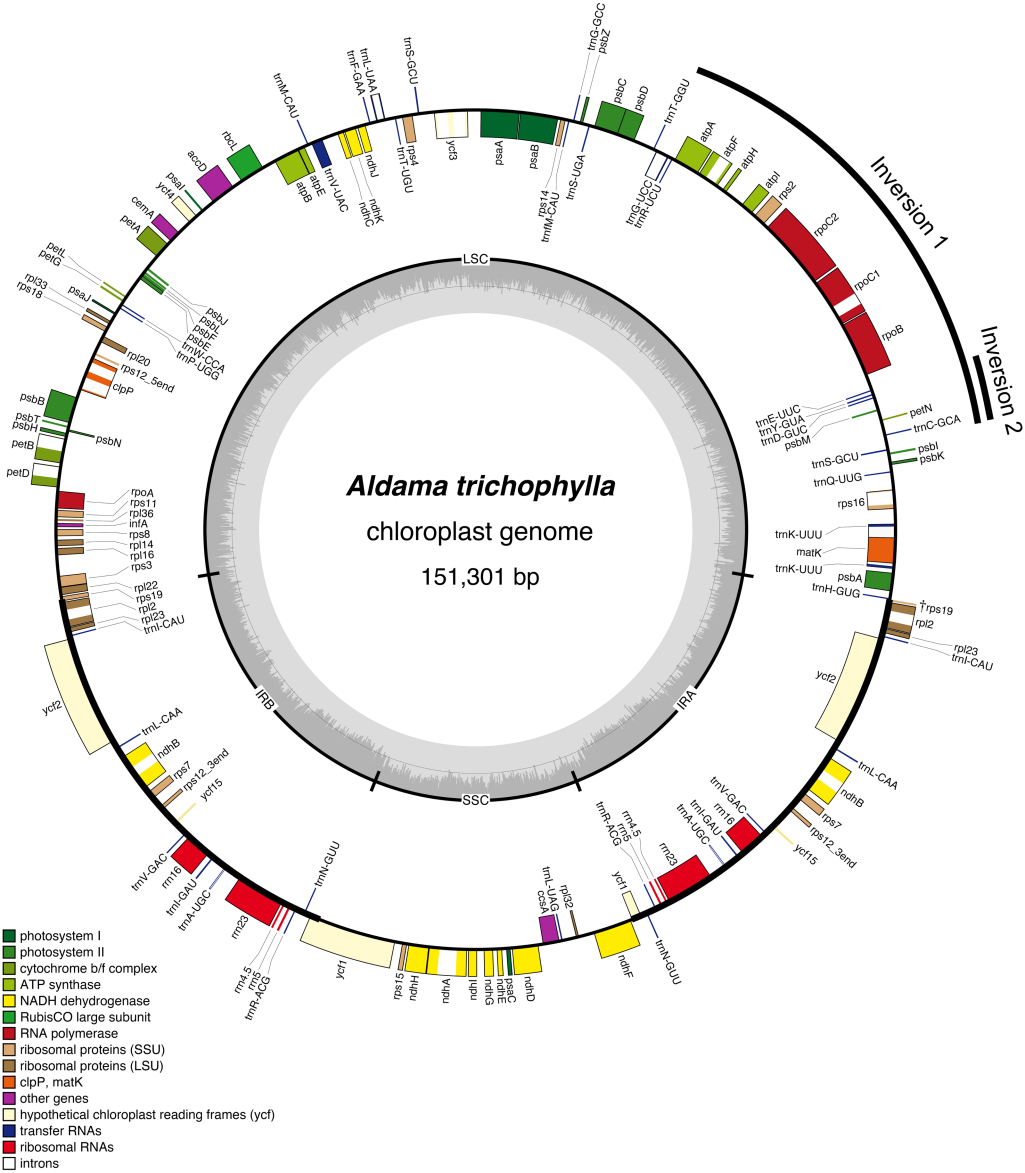
Gene map of the *Aldama trichophylla* plastome representing the genus *Aldama* and the plastomes of other five Heliantheae genera, which present the same general structure and gene content. Genes drawn inside the circle are transcribed clockwise, and those outside are transcribed counterclockwise. Genes belonging to different functional groups are colored following the legend. The darker and lighter gray in the inner circle correspond to GC content and AT content, respectively.

**Figure 2:**
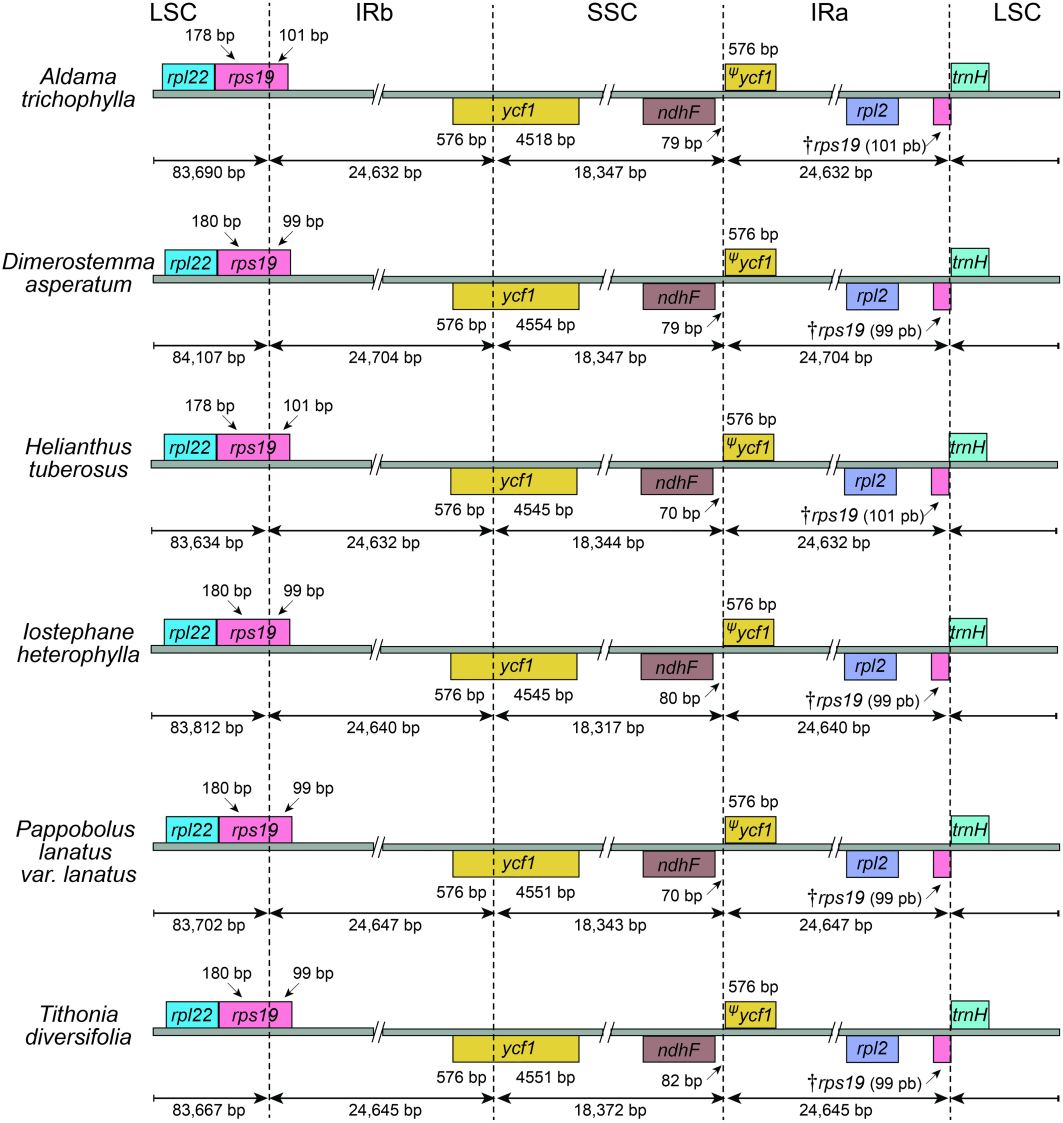
Comparisons of the Large Single Copy, Inverted Repeat a, Small Single Copy, and Inverted Repeat b boundaries among *Aldama trichophylla* and the other five Heliantheae representatives. Genes shown below the lines are transcribed reversely and those shown above are transcribed forward. Minimum and maximum sizes for the regions and structures of each plastome type that compose the borders are indicated in base pairs (bp).

The average read depth ranged between 1,431×and 5,037×(*A. nudicaulis* and *A. tuberosa*, respectively; Table S1). The 36 *Aldama* plastomes range in length from 151,221 (*A. vernonioides*) to 151,399 bp (*A. squalida*) (Table 1 and Fig. 1). The assembled plastomes include some missing data and very few degenerate bases. However, very few of these sites were found within coding regions, not significantly impacting other analyses performed here (see Tables S2 and S3). All chloroplast genomes show the typical quadripartite structure of angiosperms, consisting of a large single copy (LSC) region (83,635–83,782 bp); a small single copy (SSC) region (18,325–18,361 bp); and a pair of inverted repeat (IRs) regions (24,607–24,667 bp). The length of the plastomes of the five outgroup species from Heliantheae range from 151,242 bp (*H. tuberosus*) to 151,862 bp (*D. asperatum*), with a LSC between 83,634 bp (*H. tuberosus*) and 84,107 bp (*D. asperatum*), SSC ranging from 18,317 bp (*I. heterophylla*) to 18,372 bp (*T. diversifolia*), and a pair of IRs with length between 24,632 bp (*H. tuberosus*) and 24,704 bp (*D. asperatum*). All assembled plastomes feature a large ∼20 kb and a small, nested, ∼3 kb inversion in the LSC region, shared by other Asteraceae species (*Kim, Choi & Jansen, 2005*). The large inversion within the *Aldama* plastomes ranges from 22,350 to 22,396 bp, including 16 genes from *trnS-GCU*-*trnC-GCA* to trn*G-UCC*-*trnT-GGU*. The small inversion, with length between 3,298 and 3,328 bp, includes six genes located between *trnS-GCU-trnC-GCA* and *trnE-UUC* (Table 1 and Fig. 1). The GC content in all assembled plastomes was 37.6%. The 41 sequenced plastomes encode 113 unique genes, including 79 protein-coding genes (CDS), 30 tRNA genes, and four rRNA genes (Table 2). The IRs of all plastomes present six CDS, seven tRNA genes, and four rRNA genes, duplicated. The plastomes assembled in this study include 17 intron-containing genes, of which 14 contain one intron, while three genes contain two introns (i.e., *clpP*, *rps12*, *ycf3*) (Table 2 and Fig. 1). The *rps12* gene is trans-spliced, with the 5′end located in the LSC region and the duplicated 3′end in the IR regions. The LSC/IRs/SSC boundaries are very conserved within *Aldama* species and among other Heliantheae genera sampled in this study. The LSC/IRb boundary is within *rps19*, the IRb/SSC boundary is within *ycf1*, the SSC/IRa is between *ndhF* and a partial *ycf1* (*ψ*ycf1), and the IRa/LSC is between a truncated *rps19* (†rps19) and *trnH* (Figs. 1 and 2).

**Table 2:** Genes encoded by the *Aldama* species, *Dimerostemma asperatum, Iostephane heterophylla, Pappobolus lanatus* var. *lanatus*, and *Tithonia diversifolia* plastomes.

**Gene function**	**Gene type**	**Gene**
Self-replication	•Ribosomal RNA genes (GO:0006364)	*rrn4.5*^c^*, rrn5*^c^*, rrn16*^c^*, rrn23*^c^
	•Transfer RNA genes (GO:0061587)	*trnA-UGC*^a^^,^^c^*, trnC-GCA, trnD-GUC, trnE-UUC, trnF-GAA, trnfM-CAU, trnG-GCC, trnG-UCC*^a^*, trnH-GUG, trnI-CAU*^c^*, trnI-GAU*^a^^,^^c^*, trnK-UUU*^a^*, trnL-CAA*^c^*, trnL-UAA*^a^*, trnL-UAG, trnM-CAU, trnN-GUU*^c^*, trnP-UGG, trnQ-UUG, trnR-ACG, trnR-UCU*^c^*, trnS-GCU, trnS-GGA, trnS-UGA, trnT-GGU, trnT-UGU, trnV-GAC*^c^*, trnV-UAC*^a^*, trnW-CCA, trnY-GUA*
	•Small ribosomal subunit (GO:0015935)	*rps2, rps3, rps4, rps7*^c^*, rps8, rps11, rps12*^b^^,^^c^*, rps14, rps15, rps16*^a^*, rps18, rps19*^d^
	•Large ribosomal subunit (GO:0015934)	*rpl2*^a^^,^^c^*, rpl14, rpl16, rpl20, rpl22, rpl23*^a^^,^^c^*, rpl32, rpl33, rpl36*
	•RNA polymerase subunits (GO:0042793)	*rpoA, rpoB, rpoC1*^a^*, rpoC2*
Photosynthesis	•Photosystem I (GO:0009522)	*psaA, psaB, psaC, psaI, psaJ, ycf3*^b^*, ycf4*
	•Photosystem II (GO:0009523)	*psbA, psbB, psbC, psbD, psbE, psbF, psbH, psbI, psbJ, psbK, psbL, psbM, psbN, psbT, psbZ*
	•NADH-dehydrogenase (GO:0010258)	*ndhA*^a^*, ndhB*^a^^,^^c^*, ndhC, ndhD, ndhE, ndhF, ndhG, ndhH, ndhI, ndhJ, ndhK*
	•Cytochrome b6/f complex (GO:0017004)	*petA, petB*^a^*, petD*^a^*, petG, petL, petN*
	•ATP synthase (GO:0009544)	*atpA, atpB, atpE, atpF*^a^*, atpH, atpI*
	•Rubisco (GO:0110102)	*rbcL*
Other genes	•Translational initiator factor (GO:0006413)	*infA*
	•Maturase (GO:0006397)	*matK*
	•Protease (GO:0006508)	*clpP*^b^
	•Envelope membrane protein (GO:0009279)	*cemA*
	•Subunit of Acetil-CoA-carboxylase (GO:0003989)	*accD*
	•c-type cytochrome synthesis (GO:1903606)	*ccsA*
Unknown function	•Conserved open read frames	*ycf1*^d^*, ycf2*^c^*, ycf15*^c^


**Notes.**

a Gene with one intron.

b Gene with two introns.

c Gene duplicated.

d Gene partially duplicated.

**Figure 3:**
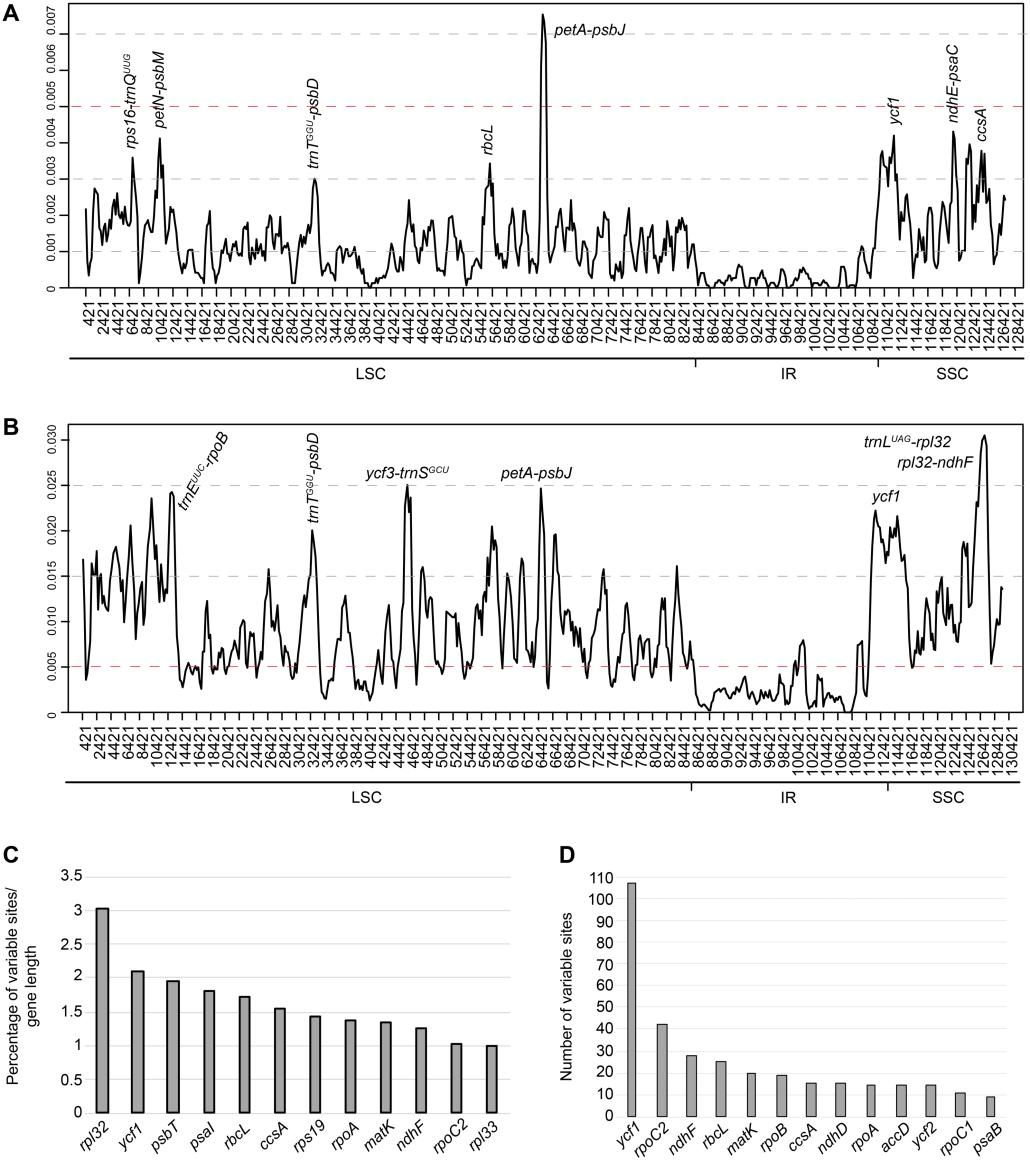
Sliding window analysis of complete plastomes. (A) within the 36 *Aldama* samples and (B) among three *Aldama* species (*Aldama anchusifolia, A. dentata, A. trichophylla*) plus outgroups of Heliantheae sequenced in this study and obtained from GenBank: *Pappobolus lanatus, Tithonia diversifolia, Helianthus tuberosus, Iostephane heterophylla, Dimerostemma asperatum, Echinaceae paradoxa* (NC_034320), *Helianthus argophyllus* (NU314500), and *Helianthus annuus* (NC_007977) (window length: 800 bp, step size: 200 bp). *X*-axis, position of the midpoint of each window; *Y*-axis, nucleotide diversity (*π*) of each window. (C–D) Twenty-seven most variable protein-coding genes within the 36 *Aldama* plastomes assembled. (C) percentage of variable sites according to gene length. (D) Number of variable sites per gene.

### Identification of variable regions

A single synteny block was retrieved from the structural analysis performed in Mauve (Fig. S1). The plastomes of *A. trichophylla* and five other Heliantheae genera show the same structure and linear order.

Levels of sequence diversity were investigated through the calculation of nucleotide variability (*π*) values within the 36 *Aldama* plastomes (Fig. 3A), among three *Aldama* species (*A. anchusifolia*, *A. dentata 2* and *A. trichophylla*) and other Heliantheae sequenced in this study or obtained from GenBank (*D. asperatum*, *E. paradoxa*, *H. annuus*, *H. argophyllus*, *H. tuberosus*, *I. heterophylla*, *P. lanatus* var. *lanatus* and *T. diversifolia*) (Fig. 3B). The *π* values within 800 bp across the plastomes range from 0 to 0.00754 (mean value of 0.00118) within *Aldama* species and from 0 to 0.0305 (mean value of 0.00860) among three *Aldama* species and the other genera, indicating that these sequences are very conserved, especially at the intrageneric level. The most variable sites within *Aldama* plastomes are within the *petA-psbJ* intergenic region (*π* between 0.00754 and 0.00613) and only three sites present *π* >0.004, which are *ndhE-psaC*, *ycf1*, and *petN-psbM* (Fig. 3A). The most variable sites among three *Aldama* species and the other genera are within the *trnL-UAG-rpl32* and *rpl32-ndhF* intergenic regions (*π* between 0.0305 and 0.02936). Further variable sites with *π* >0.020 are located within the *ycf1* gene and in other intergenic regions, such as *trnE-UUC-rpoB*, *trnT-GGU-psbD*, *ycf3-trnS-GCU* and *petA-psbJ* (Fig. 3B). In both datasets, the IRs are the most conserved regions (Fig. 3).

The alignment of the 36 complete *Aldama* plastomes (with one of the IRs removed) has 127,466 bp with 1,508 variable sites (i.e., 1.18% of variable sites). In addition, the noncoding regions are more variable (i.e., 1.66% of the intergenic regions) than the coding regions (0.73% of the protein-coding genes) (Table 3).

Among the 79 protein-coding genes, the genes with the highest percentage of variable sites are presented in Fig. 3C and Table S4. Regarding absolute numbers, the genes with more than 10 variable sites are presented in Fig. 3D and Table S4.

**Table 3:** Summary of datasets including only the 36 *Aldama* plastomes.

**Dataset**	**Length (bp)**	**Var. sites**	**Var. sites %**	**Pi sites**	**GC%**
Plastomes (LSC/IR/SSC)	127,466	1,509	1.18	814	36.7
79 genes	67,626	497	0.73	285	37.8
Intergenic regions	61,290	1,016	1.66	531	35.2
Introns	11,954	158	1.32	83	34.3

**Notes.**

Var. sites number of variable sites Var. sites% percentage of variable sites Pi sites parsimony informative sites GC% percentage of GC content

**Figure 4:**
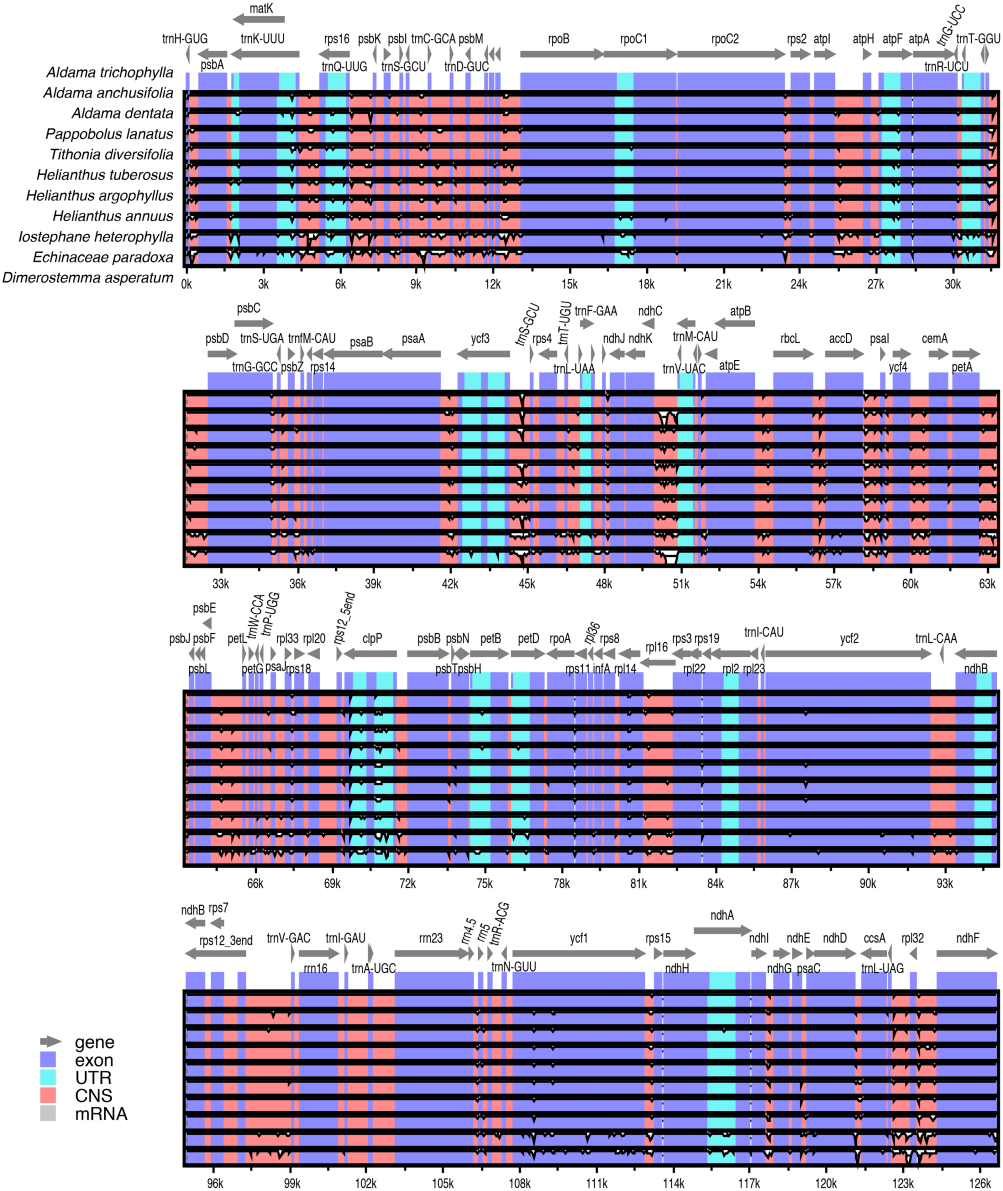
Comparison of three species of *Aldama* (i.e., *A. anchusifolia*, *A. dentata*, and *A. trichophylla*) and the five plastomes from other Heliantheae genera sequenced in this study performed in mVISTA. The plastome of *A. trichophylla* was used as reference. Dark blue blocks indicate conserved genes (CNS), light blue blocks indicate conserved introns (UTR), and red blocks indicate conserved noncoding sequences (CNS). White blocks represent regions with sequence variation among the plastomes. The vertical axis indicates sequence alignment similarity of 50–100%.

Pairwise comparisons of divergent regions within the 10 selected *Aldama* plastomes (i.e., *A. canescens*, *A. dentata* 2, *A. excelsa*, *A. filifolia*, *A. grandiflora*, *A. linearis*, *A. macrorhiza*, *A. trichophylla*, *A. rubra* and *A. veredensis*) and among three species of *Aldama* (i.e., *A. anchusifolia*, *A. dentata 2* and *A. trichophylla*) and five plastomes from other Heliantheae genera sequenced in this study were performed using mVISTA, with *A. trichophylla* as a reference (Fig. 4 and Fig. S2). Overall, the alignments reveal very low intra- and inter-generic (Fig. 4) sequence divergence across the plastomes, suggesting high degree of conservation. Noncoding regions and introns are generally more divergent than coding regions (Fig. 4 and Fig. S2).

### Selection on plastid genes

The analyses performed in Selecton to explore selection pressure on the 79 protein-coding genes within *Aldama* plastomes showed that only one gene, *rbcL*, has signatures of positive selection (adaptive selection), with only two sites out of 486 with omega values (*ω*) lower than 1, at a significance level of 0.01 for two positions (Data S3).

### SSR analyses

**Figure 5:**
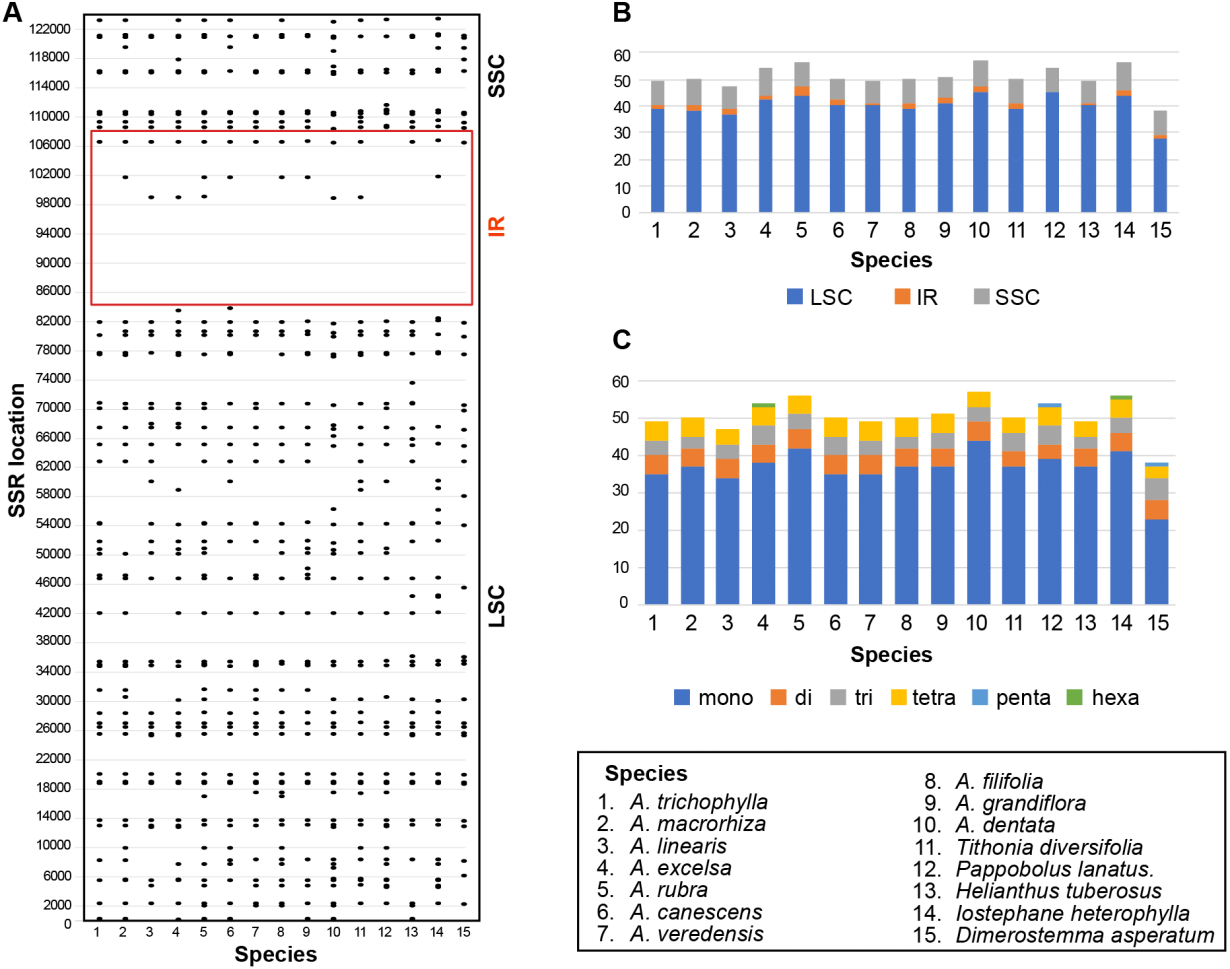
(A–B) Distribution of SSRs in the *Aldama* and the five plastomes from other Heliantheae genera sequenced in this study (IRa omitted). (C) Distribution of SSR types. .

Six kinds of repeat patterns were screened and identified using MISA in the plastomes of the 10 selected *Aldama* species listed above and the five other Heliantheae genera sequenced in this study. In *Aldama* plastomes, the total number of SSRs ranges from 47 to 57 SSRs, while in the other genera it varies from 38 (*D. asperatum*) to 56 (*I. heterophylla*) (Fig. 5A and Table S5). The most frequent SSRs are A or T mononucleotide repeats, accounting for 68–73.2% of the total SSRs in *Aldama* plastomes and for 60.5–75.5% in the other genera; G or C repeats, on the other hand, are rare (Fig. 5C and Table S5). The total number of SSR motifs in *Aldama* range between 34–44 (70–77.2%) mononucleotide repeats, 5 dinucleotide repeats in all species (8.8–10.6%), 3–5 (6–10%) trinucleotide repeats, 4–5 (7–10.2%) tetranucleotide repeats, and 0–1 (0–1.9%) hexanucleotide repeats; no pentanucleotide repeat was found (Fig. 5C and Table S5). Moreover, most (37–45) of the SSRs in *Aldama* species are located in the LSC. In *Aldama*, the IR regions include between 1–3 SSRs, while the SSC region includes between 8–10 (Fig. 5B and Table S5). In the other genera sequenced here, 28–45 of the SSRs are situated in the LSC, 0–2 in the IRs, and 8–9 in the SSC region (Figs. 5B–5C and Table S5). The density of SSRs in the LSC is somewhat similar to that found in the SSC when considering the size of each plastome region.

### Phylogenetic reconstruction

**Figure 6:**
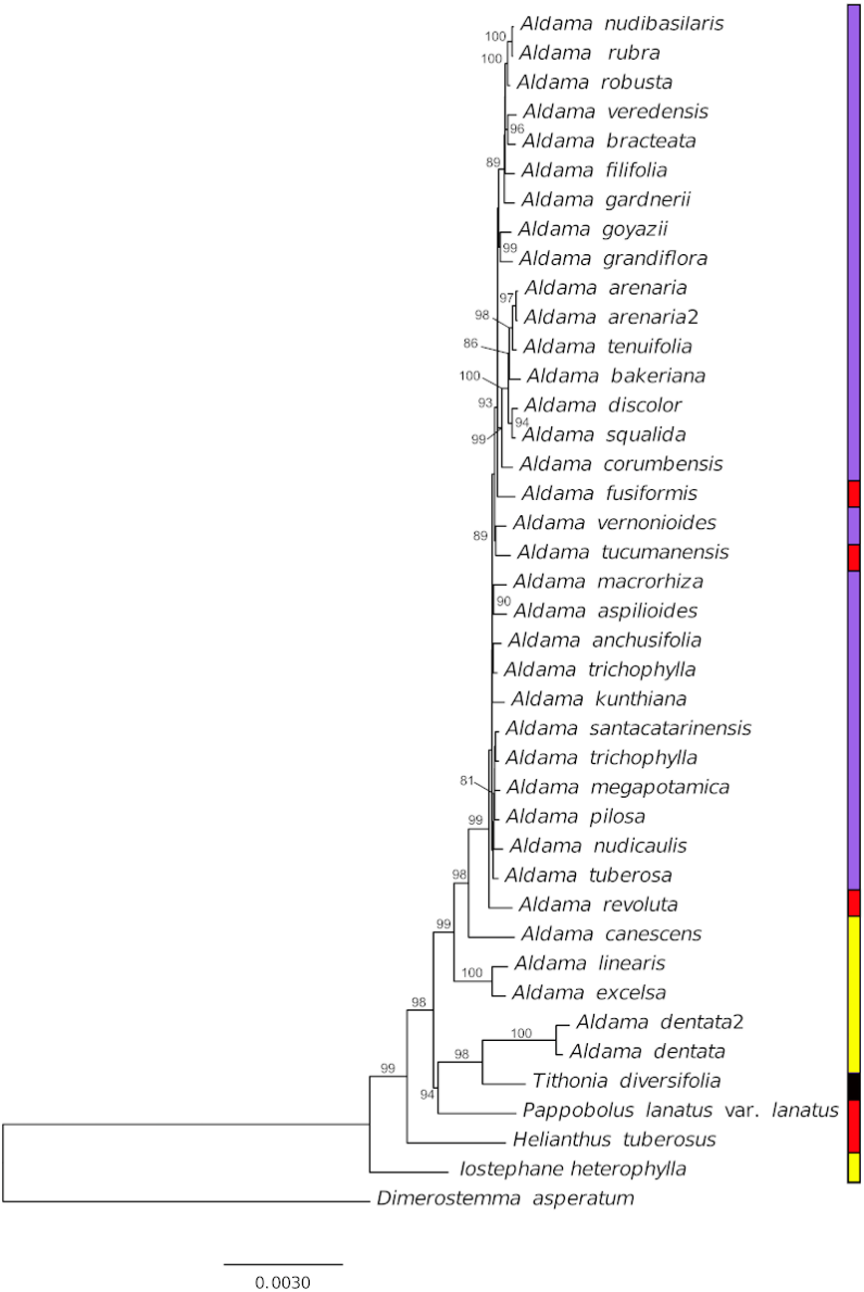
Phylogenetic relationships of 36 *Aldama* species and five species from other Heliantheae genera as outgroup based on complete chloroplast genomes, with one of the IRs removed. Branches are labeled with bootstrap values above 75% from the ML analysis. Vertical bar indicates geographic distribution, yellow, Mexico, red, Andean region, purple, Brazil and black, widespread.

The final alignment with the 41 taxa sequenced here (i.e., 36 *Aldama* species and 5 Helianthinae representatives as outgroups) is 129,218 bp long, of which 4,544 are variable sites and 1,089 are parsimony informative sites. In the ML tree (Fig. 6, bootstrap values (BS) depicted in the tree) *Aldama* is not monophyletic due to the position of the Mexican *A. dentata* as sister-group of *T. diversifolia* (BS: 98%), while all other *Aldama* species form a well-supported clade (BS: 99%). In this latter clade, Mexican and Andean species are the first to diverge (*A. linearis*, *A. excelsa*, *A. canescens* and *A. revoluta*). All Brazilian species form a strongly supported clade (BS: 99%), but internal relationships are poorly resolved due to the low nucleotide diversity found among *Aldama* plastomes, resulting in very short branches.

## Discussion

### Plastome features

In this study, we sequenced and assembled 36 complete plastomes of 33 *Aldama* species and the plastome of five other genera from the tribe Heliantheae. Even though the sequences present a number of gaps and degenerate bases, this did not affect the genetic diversity and selection analyses, as they account for a small proportion of sites in relation to the total length of each genome. In addition, coding regions were less affected by these issues, with 0.006% of missing data and zero ambiguous sites (Tables S2 and S3). The basic features of these plastomes are highly similar to those found in other Asteraceae species, including the LSC inversions that are present in nearly all members of the family. Our results show that the plastomes are highly conserved in size, GC content, number of genes and gene order (*Wang et al., 2015; Salih et al., 2017*). Size variation between the smallest (*A. vernonioides*) and largest (*A. squalida*) *Aldama* plastomes is very low (178 bp) (Table 1). This is similar to the variation found between the plastomes of different species of *Atractylodes* (60 bp; *Wang et al., 2020)*, *Echinacea* (98 bp; *Zhang et al., 2017)*, *Dendrosenecio* (59 bp; *Gichira et al., 2019)*, *Dolomiaea* (179 bp; *Shen et al., 2020)* and *Senecio* (209 bp) (*Gichira et al., 2019*), but greatly inferior to the variation found between 20 *Saussurea* species (1,148 bp; *Zhang et al., 2019)*. Among genera in Heliantheae, *D. asperatum,* from subtribe Ecliptinae, presents the largest genome, exceeding the smallest one (*A. vernonioides*) by only 641 bp. Among members of subtribe Helianthinae, the size difference is even smaller (274 bp) (Table 1). Higher variation in size was found among members of other tribes, such as Cardueae (1,138 kb; Zhang et al., 2019) and Senecioneae (865 bp; *Gichira et al., 2019*). No significant expansions of the IRs (60 bp) or contractions of the LSC (147 bp) have been detected among *Aldama* species, or among the Heliantheae genera (IRs: 97 bp.; LSC: 472 bp) (Table 1 and Fig. 1). Expansions of the IRs and contractions of the LSC are frequent in Asterids (e.g., *Downie & Jansen, 2015; Firetti et al., 2017; Thode & Lohmann, 2019*), but were not observed at the generic level in our results or in other studies in Asteraceae (*Zhang et al., 2017; Cho et al., 2019; Gichira et al., 2019; Shahzadi et al., 2019; Shen et al., 2020; Wang et al., 2020; Zhang et al., 2019*), except for a recent study in the genus *Aster* (*Tyagi et al., 2020*). The IR/SC boundaries are highly conserved in all *Aldama* and Heliantheae genera sequenced in this study (Fig. 2). Positions of these boundaries are very constant in Asteraceae (*Wang et al., 2015*). Some variation has been found at the IRb/SSC boundary in the tribe Cardueae and in *Aster* (within *ycf1* or between the genes *ycf1* and *ndhF*) (*Zhang et al., 2019; Tyagi et al., 2020*), but we observed conserved borders within *ycf1* in *Aldama* and the Heliantheae genera.

In all plastomes studied here (33 species of *Aldama* and five Heliantheae genera), 18 genes are duplicated. Like in nearly all Asteraceae, there are seven tRNA genes and four rRNA genes located in the IR regions (*Wang et al., 2015*). Similar to all Angiosperms, the *rps12* gene was found to be trans-spliced (one of its exons located in the LSC region and the other duplicated in the IRs) (*Howe et al., 2003*). Duplication of the *trnF-GAA* gene has probably occurred several times in the evolutionary history of Asteraceae, in different subfamilies (Carduoideae, Cichorioideae and Asteroideae) (*Salih et al., 2017*). We did not detect the *trnF-GAA* gene duplication in the *Aldama* species and Heliantheae genera assembled here, as previously seen in other taxa of Heliantheae, such as *H. annuus* (*Timme et al., 2007*) and *Echinacea* (*Zhang et al., 2017*). Nonetheless, this duplication was reported for *Lasthenia burkeyi* (Heliantheae, subtribe Madieae) (*Walker, Zanis & Emery, 2014*). Three other frequent duplications in Asteraceae (*ndhB*, *rpl2* and *rpl23*; all located in the IR regions) (*Timme et al., 2007; Salih et al., 2017; Zhang et al., 2017; Cho et al., 2019; Tyagi et al., 2020; Wang et al., 2020*) were observed in our results. Another frequently duplicated gene in Asteraceae, *ycf15*, was detected in *Aldama* and the Heliantheae genera sequenced here, as observed in *H. annuus* (*Timme et al., 2007*), *Echinacea* (*Zhang et al., 2017*) and *Aster* (*Tyagi et al., 2020*), but its complete absence was reported in some species of *Chrysanthemum* (tribe Anthemideae) (*Wang et al., 2015*) and *Guizotia abyssinica* (tribe Millerieae) (*Dempewolf et al., 2010*) (Table 2).

The largest plastid gene in the Angiosperms, *ycf2,* displays a ∼456 bp deletion in *Helianthus* species, which does not seem to affect its functionality (Timme et al., 2007; Zhang et al., 2019), even though its vital function remains unknown (*Drescher et al., 2000*). Such partial deletion in *ycf2* has also been observed in Poaceae (*Millen et al., 2001; Matsuoka et al., 2002*). We found a slightly smaller deletion in all *Aldama* species, with size varying from 441 bp (most species) to 447 bp (in two species), similar to the values found in species of *Helianthus* (Zhang et al., 2019). The same 441 bp deletion in *ycf2* was found in the three taxa of subtribe Helianthinae (*I. heterophylla*, *P. lanatus* var. *lanatus* and *T. diversifolia*) and in *Dimerostemma,* from subtribe Ecliptinae, even though this deletion is absent in the plastome of another member of this subtribe, *Eclipta prostata* (*Park et al., 2016*). The deletion in *ycf2* is absent in species of *Echinacea,* from subtribe Zinniinae (*Zhang et al., 2017*). These results indicate that the deletion in *ycf2* is not an unique event in the evolutionary history of Heliantheae, as it is not restricted to the derived taxa of subtribe Helianthinae (*Schilling & Jansen, 1989*), also occurring in subtribe Ecliptinae. However, the current knowledge about phylogenetic relationships among Heliantheae subtribes (*Panero, 2007*) is insufficient to understand the evolution of *ycf2* within the tribe.

The conservation of plastome features has been recorded in several Angiosperm groups (*Cai et al., 2015; Reginato et al., 2016; Sun et al., 2019; Yan et al., 2019*) and our results corroborate that plastomes are highly conserved in Asteraceae, even at the generic or subtribal level, in size, GC content, number of genes and gene order (*Wang et al., 2015; Salih et al., 2017; Shen et al., 2020*).

### Variable regions

The plastomes of *Aldama* and other genera of Heliantheae studied here display very low intrageneric and intergeneric sequence divergence. Noncoding regions and introns are slightly more divergent than coding regions and the IRs are the most conserved regions (Figs. 3 and 4 and Fig. S1–Fig. S2). Among *Aldama* plastomes, the *petA-psbJ* intergenic region is the most variable locus and only three other regions have some significant variability: *ndhE-psaC*, *petN-psbM* and *ycf1* (Fig. 4A). The intergenic region *petA-psbJ* is rarely used in phylogenetic inferences (e.g., Huang et al., 2004; Techaprasan et al., 2010), and has never been used in Asteraceae. Shaw et al. (2007) and Shaw et al. (2014) pointed out this region as one of the most variable within the chloroplast genome across angiosperm lineages, including among closely related species of *Aster* (*Tyagi et al., 2020*). Dong et al. (2012) suggested it as a potential candidate for DNA barcoding and phylogenies in lower taxonomic levels. The noncoding region *ndhE-psaC* has never been used in phylogenetic inference and has only been cited as a hypervariable region in monocots (*Lu, Li & Qiu, 2017; Santana Lopes et al., 2018; Cui et al., 2019*). The *petN-psbM* intergenic spacer was reported as a variable region in Asteraceae once, in *Echinacea*, another genus of Heliantheae (*Zhang et al., 2017*), and Shaw et al. (2014) described it as a potential variable region in specific groups (e.g., Fonseca & Lohmann, 2017). In a similar way to other intergenic regions that evolve rapidly, minute inversions have already been noted in *petN-psbM*, requiring some care when aligning it (*Kim & Lee, 2005; Dong et al., 2012*).

While coding regions normally accumulate genetic differences more slowly than non-coding regions (*Fazekas et al., 2008; Lahaye et al., 2008*), one exception is the gene *ycf1*, which evolves at a faster pace (*Dong et al., 2012; Shaw et al., 2014*). This region is frequently cited as one of the most variable in Asteraceae plastomes (*Wang et al., 2015; Zhang et al., 2017; Cho et al., 2019; Zhang et al., 2019; Shen et al., 2020; Wang et al., 2020*) but seems to have been used as a marker only once, in a phylogenetic study within the tribe Astereae (*Costa, 2014*). Nonetheless, *ycf1* was successfully used in phylogenetic inferences of other angiosperm groups, such as Fabaceae (e.g., Dastpak et al., 2018) and orchids (e.g., *Neubig et al., 2009)*. Dong et al. (2015) concluded that *ycf1* can serve as a core barcode for land plants. Regarding the percentage of variable sites (in proportion to sequence length), *ycf1* is the most variable coding region, followed by *rpl32* (Fig. 3C and Table S4). When considering absolute numbers, *rpoC2* has the highest number of sites, followed by *ycf1* (Fig. 3D and Table S4). The *rpl32* gene is rarely cited in Asteraceae as a variable region (*Timme et al., 2007*) and, due to its small size (165 bp), the region has never been used in phylogenetic inferences. The *rpo* genes, which code for RNA polymerase subunits, are relatively rapidly evolving genes (*Krawczyk & Sawicki, 2013*). Other studies in Asteraceae (*Salih et al., 2017; Cho et al., 2019*) also pointed out *rpoC2* as a variable region and Wang et al. (2015) found some species-specific editing sites in *Lactuca sativa,* suggesting lineage specific evolutionary features of RNA editing for that region. While *rpoC2* has not been used for phylogenetic inference in Asteraceae, its utility was demonstrated in other Angiosperm families like Poaceae (*Peterson, Romaschenko & Arrieta, 2016*) and Verbenaceae (*Marx et al., 2010*). In addition, Logacheva et al. (2017) concluded that *rpo* genes might be very useful for phylogenetic reconstructions. As a whole, the nucleotide composition among *Aldama* species is very conserved, with a percentage of variable sites (1.18%) somewhat lower than that found in the *Espeletia* species complex (2.46%; Pouchon et al., 2018), among species of *Saussurea* (4.07%; *Zhang et al., 2019)* and in Hawaiian species of *Bidens* (4.8%; *Knope et al., 2020)*, but similar to *Echinacea* (<1%; *Zhang et al., 2017)*.

When comparing the plastomes of the five genera of Heliantheae with three species of *Aldama*, the variability found is a little bit higher. The most variable regions are the gene *ycf1* and two intergenic regions, *trnL-UAG-rpl32* and *rpl32-ndhF* (Fig. 3B). These two intergenic regions have been widely reported as the most divergent regions across Angiosperm lineages (*Shaw et al., 2007; Dong et al., 2012; Shaw et al., 2014*), especially *rpl32-ndhF* in Asteraceae (*Timme et al., 2007; Salih et al., 2017; Zhang et al., 2017; Gichira et al., 2019, Shahzadi et al., 2019; Zhang et al., 2019; Nie et al., 2020*) and less frequently *trnL-UAG-rpl32* (*Timme et al., 2007; Cho et al., 2019; Shahzadi et al., 2019; Shen et al., 2020*). Numerous phylogenies in Asteraceae used *trnL-UAG-rpl32* as a molecular marker (e.g., *Freire et al., 2015; Loeuille, Keeley & Pirani, 2015; Loeuille et al., 2015; Padin, Calviño & Ezcurra, 2015; Soto-Trejo et al., 2015; Jara-Arancio, Vidal & Arroyo, 2018; Oberprieler et al., 2019*), sometimes associated with *ndhF-rpl32* (*Dillenberg & Kadereit, 2013; Steffen, Dillenberger & Kadereit, 2016; Gutiérrez-Larruscain et al., 2018; Gerschwitz-Eidt & Kadereit, 2019*).

### Signature of positive selection in plastid genes

The present study indicates that among the 79 protein-coding genes within *Aldama*, only the gene *rbcL* is significantly under positive selection (*ω* >1). It encodes for the larger subunit of the enzyme rubisco, which catalyzes the first step in photosynthetic assimilation of inorganic carbon and photorespiration (*Spreitzer & Salvucci, 2002; Feller, Anders & Mae, 2008; Bathellier et al., 2018; Erb & Zarzycki, 2018; Pottier, Gilis & Boutry, 2018*). Purifying selection has been frequently reported in Asteraceae for most of the plastidial protein-coding genes (*Shahzadi et al., 2019; Zhang et al., 2019*). The gene *ndhF* was shown to be under positive selection in *Aster* (*Tyagi et al., 2020*) and in *Dolomiaea* (*Shen et al., 2020*), along with *atpA* and *rbcL* in the latter*.* Zhang et al. (2019) found positive selection for *ycf2* in *Helianthus*, but contrary to the expectation, we did not detect a positive selection signature in *Aldama*. The extremely low nucleotide diversity observed among chloroplast sequences might compromise our capacity to detect that signal.

Positive selection for *rbcL* is widespread among land plants (*Kapralov & Filatov, 2007; Yao et al., 2019; Shen et al., 2020*) and several studies have demonstrated that positive selection on specific amino-acids in rubisco is linked to plant adaptation to different environmental stresses, facilitating adaptive radiation into diverse ecological niches (*Hermida-Carrera et al., 2017*). Examples include adaptations to dry or wet climates (*Kapralov & Filatov, 2006; Galmés et al., 2014*), to high altitude environments (*Hu et al., 2015*) and in transitions from C_3_ to C_4_ photosynthesis (*Christin et al., 2008; Kapralov, Smith & Filatov, 2012; Hermida-Carrera et al., 2020*), including in *Flaveria* (Asteraceae, tribe Tageteae) (*Kapralov et al., 2011*).

### SSRs in *Aldama* plastomes

Single Sequence Repeats (SSRs) are often observed in chloroplast genomes, being useful markers for evolutionary studies in lower taxonomic levels, such as population genetics (*Avise, 1994; Ebert & Peakall, 2009; Qi et al., 2016; Yu et al., 2017*). The SSRs most frequently found in chloroplast genomes are poly-A tails, usually also being the ones showing more variability. As NGS methods became cheaper and more popular, the *in silico* mining of microsatellite has become more common, with the advantage of easily separating organellar repeats from nuclear repeats when mapping against a reference genome. Recent papers dealing with the development of SSR markers in Asteraceae from chloroplast genomes have shown very little variability in the tested loci, especially when excluding mononucleotide repeats (*Siniscalchi et al., 2019; Thapa, Bayer & Mandel, 2019*).

The SSRs regions presented here can be used as basis for future population genetic studies involving *Aldama*, although their variability needs to be validated in different populations (Figs. 6A–6C). The fact that several repeat regions are present in more than one species is a good indication that they could be easily transferable across the genus, being useful for multi-species studies. While several Brazilian species complexes within *Aldama* have been the focus of detailed anatomical and phytochemical studies (*Bombo et al., 2012; Bombo et al., 2014; Silva & Appezzato-da Glória, 2014; Bombo, Filartiga & Appezzato-Da-Glória, 2016; Filartiga et al., 2016; Filartiga et al., 2017*), populational studies based on these SSRs would greatly improve the understanding of population dynamics and microevolutionary processes in these species.

### Phylogenetic hypothesis

Our phylogenetic analysis indicates that in the current circumscription, *Aldama* is not monophyletic, as *A. dentata* is more closely related to other Mexican and Andean genera (*Pappobolus* and *Tithonia*) than to the rest of the *Aldama* species (Fig. 6). This result is in agreement with plastidial restriction site analysis (*Schilling & Panero, 1996a*) but not with ribosomal ITS and ETS phylogenetic analyses (*Schilling & Panero, 2011*). More studies are necessary to understand if this incongruence between plastome and nuclear data is a consequence of an evolutionary history of hybridization and chloroplast capture in the Helianthinae (*Schilling & Panero, 1996b*) or an artefact of the low taxonomic sampling of Mexican Helianthinae in our analysis.

The Mexican *Aldama* species are early diverging lineages, while all South American members of *Aldama* form a strongly supported clade. Nonetheless, the relationships between the Brazilian and Andean species are poorly resolved due to the extremely low nucleotide diversity (Fig. 6).

## Conclusions

The analyses comparing 36 *Aldama* plastomes and the plastomes of five other genera belonging to Heliantheae provided significant new understanding about Asteraceae plastome evolution and structure. Within *Aldama* and among the other genera compared here, plastomes show very similar lengths, number of genes, and boundaries between the regions. The nucleotide variability was extremely low at the intrageneric level. Our results show that plastomes may be extremely conserved and not suited for phylogenetic analysis at the intrageneric level. Our results also shed light on a more complex evolution of the large deletion in the *ycf2* gene in Heliantheae, which was probably gradual and involved multiple events. Signals of positive selection detected in the *rbcL* gene in *Aldama* raise an interesting question on the adaptative radiation of the genus that instigates further investigation. Finally, the obtained data bring powerful information for further studies aiming at a robust phylogenetic circumscription of *Aldama*, indicating regions that are more variable and SSR markers useful for population genetics studies.

##  Supplemental Information

10.7717/peerj.10886/supp-1 Supplemental Information 1 Coverage across each chloroplast assembly, number are number of reads

10.7717/peerj.10886/supp-2 Supplemental Information 2 Summary of amount on missing data and degenerate bases in the alignments Length (bp): alignment length, Total number of sites: length of alignment x number of plastomes (36), Missing sites: number of missing sites, % Missing:percentage of missing sites, % Ambiguous sites: percentage of ambiguous sites.

10.7717/peerj.10886/supp-3 Supplemental Information 3 Summary of amount on missing data and degenerate bases in the five coding regions with missing data Length (bp): alignment length, Total number of sites: length of alignment x number of plastomes (36), Missing sites: number of missing sites, % Missing:percentage of missing sites, % Ambiguous sites: percentage of ambiguous sites.

10.7717/peerj.10886/supp-4 Supplemental Information 4 Summary of the 79 protein-coding gene alignments extracted from the 38 Aldama plastomes Alignment length (bp), length of alignment translated in amino acids, number of variable sites, percentage of variable sites, parsimony informative sites (Pi sites), and percentage of GC content (GC%) for the nucleotide alignments.

10.7717/peerj.10886/supp-5 Supplemental Information 5 Results from the distribution of SSRs in all plastomes of the *Aldama* species studied here and five other Heliantheae genera

10.7717/peerj.10886/supp-6 Supplemental Information 6 Synteny detected in the plastomes from *Aldama trichophylla* and other Heliantheae genera sequenced here The red block indicates a single synteny block showing that the plastomes from these six genera are very conserved.

10.7717/peerj.10886/supp-7 Supplemental Information 7 Comparison within 10 selected *Aldama* plastomes (i.e., *Aldama trichophylla, A. macrorhiza, A. linearis, A. excelsa, A. rubra, A. canescens, A. veredensis, A. filifolia, A. grandiflora, A. dentata 2*) performed in mVISTA The plastome of *A. trichophylla* was used as reference. Dark blue blocks indicate conserved genes (CNS), light blue blocks indicate conserved introns (UTR), and red blocks indicate conserved noncoding sequences (CNS). White blocks represent regions with sequence variation among the plastomes. The vertical axis indicates sequence alignment similarity of 50–100%.

10.7717/peerj.10886/supp-8 Supplemental Information 8 Sample geographical information

10.7717/peerj.10886/supp-9 Supplemental Information 9 Sequences of the RNA baits used for plastome capture

10.7717/peerj.10886/supp-10 Supplemental Information 10 ratio of synonymous (Ks; silent) and non-synonymous (Ka; amino-acid altering) substitutions in the codon alignments

10.7717/peerj.10886/supp-11 Supplemental Information 11 DNA sequences of Aldama and outgroup taxa

## Additional Information and Declarations

### Data Availability

The following information was supplied regarding data availability: Raw data are available in Table 1. All plastome sequences are available in the Supplemental Files and at GenBank: Data is available at GenBank: MN337902, MN337903, MN337904, MN337905, MN337906, MN337907, MN337908, MN337909, MN337910, MN356024, MN356025, MN356026,  MN337890, MN337891, MN337892, MN337893, MN337894, MN337895, MN337896, MN337897, MN337898, MN337899, MN337900, MN337901, MN356027, MN356028, MN356029, MN356030, MN356031, MN356032, MN337911, MN311247, MN356033, MN356034, MN356035, MN356036, MT700540, MT700541, MT700542, MT700543, MT700544.
