# Supplementary material for: Extremely low nucleotide diversity among thirty-six new chloroplast genome sequences from Aldama (Heliantheae, Asteraceae) and comparative chloroplast genomics analyses with closely related genera
Source: PeerJ. 2021 Feb 24;9:e10886. doi: 10.7717/peerj.10886 (PMC7912680; doi:10.7717/peerj.10886)
Supplement: Supplemental Information 1 [file peerj-09-10886-s001.docx]

**Supplemental Table 1.** Coverage across each chloroplast assembly, number are number of reads.

| Species | Min. | Median | Mean | Max. |
| --- | --- | --- | --- | --- |
| *Aldama anchusifolia* | 0 | 4165 | 4160 | 8001 |
| *Aldama arenaria 1* | 0 | 2798 | 2842 | 7195 |
| *Aldama arenaria 2* | 0 | 4171 | 4267 | 8000 |
| *Aldama aspilioides* | 0 | 4326 | 4325 | 8002 |
| *Aldama bakeriana* | 0 | 3242 | 3252 | 7996 |
| *Aldama bracteata* | 0 | 4291 | 4283 | 8002 |
| *Aldama canescens* | 1 | 5180 | 4865 | 8004 |
| *Aldama corumbensis* | 0 | 2307 | 2384 | 7293 |
| *Aldama dentata 1* | 0 | 4391 | 4327 | 8001 |
| *Aldama dentata 2* | 0 | 2233 | 2211 | 5973 |
| *Aldama discolor* | 0 | 4189 | 4068 | 8000 |
| *Aldama excelsa 1* | 1 | 4278 | 4274 | 8002 |
| *Aldama excelsa 2* | 0 | 4258 | 4218 | 8002 |
| *Aldama filifolia* | 0 | 3860 | 3881 | 8001 |
| *Aldama fusiformis* | 4 | 3850 | 3940 | 8001 |
| *Aldama gardnerii* | 1 | 2066 | 2069 | 5718 |
| *Aldama goyazii* | 4 | 2877 | 2844 | 6647 |
| *Aldama grandiflora* | 2 | 3550 | 3518 | 7999 |
| *Aldama kunthiana* | 4 | 3712 | 3712 | 7999 |
| *Aldama linearis* | 1 | 4171 | 4198 | 8001 |
| *Aldama macrorhiza* | 0 | 2595 | 2561 | 6077 |
| *Aldama megapotamica* | 3 | 1911 | 1907 | 4333 |
| *Aldama nudibasilaris* | 5 | 3545 | 3590 | 8000 |
| *Aldama nudicaulis* | 0 | 1405 | 1431 | 4050 |
| *Aldama pilosa* | 1 | 3084 | 3125 | 7989 |
| *Aldama revoluta* | 2 | 3336 | 3411 | 7998 |
| *Aldama robusta* | 1 | 3913 | 3903 | 7999 |
| *Aldama rubra* | 2 | 2935 | 2986 | 7995 |
| *Aldama santacatarinensis* | 7 | 2572 | 2572 | 6174 |
| *Aldama squalida* | 3 | 2745 | 2788 | 7464 |
| *Aldama tenuifolia* | 3 | 3184 | 3263 | 8000 |
| *Aldama trichophylla* 1 | 5 | 2514 | 2539 | 6567 |
| *Aldama trichophylla* 2 | 0 | 2933 | 2863 | 6864 |
| *Aldama tuberosa* | 6 | 5477 | 5037 | 8002 |
| *Aldama tucumanensis* | 0 | 4340 | 4246 | 8000 |
| *Aldama veredensis* | 1 | 3201 | 3291 | 8000 |
| *Aldama vernonioides* | 3 | 2883 | 2913 | 6942 |
| *Dimerostemma asperatum* | 0 | 3562 | 3521 | 8000 |
| *Helianthus tuberosus* | 3 | 4309 | 4212 | 8000 |
| *Iostephane heterophylla* | 0 | 4234 | 4208 | 8001 |
| *Pappobolus lanatus* var. *lanatus* | 0 | 3574 | 3557 | 7996 |
