# Supplementary material for: Extremely low nucleotide diversity among thirty-six new chloroplast genome sequences from Aldama (Heliantheae, Asteraceae) and comparative chloroplast genomics analyses with closely related genera
Source: PeerJ. 2021 Feb 24;9:e10886. doi: 10.7717/peerj.10886 (PMC7912680; doi:10.7717/peerj.10886)
Supplement: Supplemental Information 2 — Length (bp): alignment length, Total number of sites: length of alignment x number of plastomes (36), Missing sites: number of missing sites, % Missing:percentage of missing sites, % Ambiguous sites: percentage of ambiguous sites. [file peerj-09-10886-s002.docx]

**Supplemental Table S2.** Summary of amount on missing data and degenerate bases in the alignments.

| **Dataset** | **Length (bp)** | **Total number of sites [alignment length x number of plastomes (36)]** | **Missing sites** | **% Missing** | **Degenerate bases** | **% Ambigous sites** |
| --- | --- | --- | --- | --- | --- | --- |
| Plastomes (LSC/IR/SSC) | 127,466 | 4,588,776 | 5,859 | 0.128 | 1,269 | 0.028 |
| 79 genes | 67,626 | 2,434,536 | 149 | 0.006 | 0 | 0.000 |
| Intergenic regions | 61,290 | 2,206,440 | 5,800 | 0.263 | 1,464 | 0.066 |
| Introns | 11,954 | 430,344 | 649 | 0.151 | 118 | 0.027 |

**Note**: Length (bp): alignment length, Total number of sites: length of alignment x number of plastomes (36), Missing sites: number of missing sites, % Missing: percentage of missing sites, % Ambiguous sites: percentage of ambiguous sites.
