# Supplementary material for: Extremely low nucleotide diversity among thirty-six new chloroplast genome sequences from Aldama (Heliantheae, Asteraceae) and comparative chloroplast genomics analyses with closely related genera
Source: PeerJ. 2021 Feb 24;9:e10886. doi: 10.7717/peerj.10886 (PMC7912680; doi:10.7717/peerj.10886)
Supplement: Supplemental Information 3 — Length (bp): alignment length, Total number of sites: length of alignment x number of plastomes (36), Missing sites: number of missing sites, % Missing:percentage of missing sites, % Ambiguous sites: percentage of ambiguous sites. [file peerj-09-10886-s003.docx]

**Supplemental Table S3.** Summary of amount on missing data and degenerate bases in the five coding regions with missing data.

| **Coding regions with missing data** | **Length (bp)** | **Total number of sites** | **Missing sites** | **% Missing** | **Degenerate bases** | **% Ambigous sites** |
| --- | --- | --- | --- | --- | --- | --- |
| *clpP* | 591 | 21,276 | 4 | 0.0188 | 0 | 0 |
| *matK* | 1,503 | 54,108 | 15 | 0.0277 | 0 | 0 |
| *ndhH* | 1,182 | 42,552 | 9 | 0.0212 | 0 | 0 |
| *rpoB* | 3,183 | 114,588 | 2 | 0.0017 | 0 | 0 |
| *ycf1* | 5,100 | 183,600 | 18 | 0.0098 | 0 | 0 |

**Note**: Length (bp): alignment length, Total number of sites: length of alignment x number of plastomes (36), Missing sites: number of missing sites, % Missing: percentage of missing sites, % Ambiguous sites: percentage of ambiguous sites.
