# Supplementary material for: Extremely low nucleotide diversity among thirty-six new chloroplast genome sequences from Aldama (Heliantheae, Asteraceae) and comparative chloroplast genomics analyses with closely related genera
Source: PeerJ. 2021 Feb 24;9:e10886. doi: 10.7717/peerj.10886 (PMC7912680; doi:10.7717/peerj.10886)
Supplement: Supplemental Information 4 — Alignment length (bp), length of alignment translated in amino acids, number of variable sites, percentage of variable sites, parsimony informative sites (Pi sites), and percentage of GC content (GC%) for the nucleotide alignments. [file peerj-09-10886-s004.docx]

**Supplemental Table 2.** Summary of the 79 protein-coding gene alignments extracted from the 36 *Aldama* plastomes: alignment length (bp), length of alignment translated in amino acids, number of variable sites, percentage of variable sites, parsimony informative sites (Pi sites), and percentage of GC content (GC%) for the nucleotide alignments.

| **Gene** | **Length (bp)** | **Length translated** | **Var. sites** | **% variable** | **Pi sites** | **GC%** |
| --- | --- | --- | --- | --- | --- | --- |
| **rpl32** | 165 | 55 | 5 | 3.03 | 4 | 34.6 |
| **ycf1** | 5,100 | 1,700 | 107 | 2.10 | 68 | 29.8 |
| **psbT** | 103 | 34 | 2 | 1.94 | 1 | 32.3 |
| **psaI** | 111 | 37 | 2 | 1.80 | 1 | 36.9 |
| **rbcL** | 1,458 | 486 | 25 | 1.71 | 18 | 43.6 |
| **ccsA** | 975 | 325 | 15 | 1.54 | 8 | 31.1 |
| **rps19** | 279 | 93 | 4 | 1.43 | 2 | 34.4 |
| **rpoA** | 1,029 | 343 | 14 | 1.36 | 7 | 35.4 |
| **matK** | 1,503 | 501 | 20 | 1.33 | 10 | 33.5 |
| **ndhF** | 2,232 | 744 | 28 | 1.25 | 19 | 32.3 |
| **rpoC2** | 4,146 | 1,382 | 42 | 1.01 | 24 | 37.9 |
| **rpl33** | 201 | 67 | 2 | 1.00 | 1 | 39.8 |
| **ndhD** | 1,521 | 507 | 15 | 0.99 | 7 | 36.3 |
| **ndhE** | 306 | 102 | 3 | 0.98 | 1 | 31.7 |
| **rpl16** | 411 | 137 | 4 | 0.97 | 0 | 42.1 |
| **accD** | 1,443 | 481 | 14 | 0.97 | 7 | 36.0 |
| **rps3** | 657 | 219 | 6 | 0.91 | 2 | 34.4 |
| **petG** | 114 | 38 | 1 | 0.88 | 1 | 36.0 |
| **psbF** | 120 | 40 | 1 | 0.83 | 1 | 42.5 |
| **ndhC** | 363 | 121 | 3 | 0.83 | 1 | 34.4 |
| **psbJ** | 123 | 41 | 1 | 0.81 | 0 | 41.5 |
| **atpI** | 744 | 248 | 6 | 0.81 | 4 | 39.1 |
| **ndhG** | 531 | 177 | 4 | 0.75 | 1 | 36.0 |
| **rps16** | 267 | 89 | 2 | 0.75 | 1 | 37.9 |
| **rps8** | 405 | 135 | 3 | 0.74 | 1 | 36.3 |
| **rps2** | 711 | 237 | 5 | 0.70 | 2 | 37.7 |
| **ndhH** | 1,182 | 394 | 8 | 0.68 | 5 | 38.2 |
| **rps4** | 606 | 202 | 4 | 0.66 | 2 | 40.4 |
| **rps14** | 303 | 101 | 2 | 0.66 | 2 | 40.9 |
| **rps18** | 306 | 102 | 2 | 0.65 | 1 | 34.0 |
| **petA** | 963 | 321 | 6 | 0.62 | 4 | 40.4 |
| **ndhI** | 501 | 167 | 3 | 0.60 | 1 | 34.6 |
| **rpoB** | 3,183 | 1,061 | 19 | 0.60 | 9 | 38.6 |
| **ndhK** | 678 | 226 | 4 | 0.59 | 2 | 38.4 |
| **psbA** | 1,062 | 354 | 6 | 0.56 | 4 | 42.1 |
| **psbD** | 1,062 | 354 | 6 | 0.56 | 4 | 42.7 |
| **ycf4** | 555 | 185 | 3 | 0.54 | 2 | 38.2 |
| **rpoC1** | 2,070 | 690 | 11 | 0.53 | 5 | 37.6 |
| **rpl20** | 381 | 127 | 2 | 0.52 | 2 | 36.7 |
| **atpA** | 1,527 | 509 | 8 | 0.52 | 5 | 40.6 |
| **ndhA** | 1,093 | 364 | 5 | 0.46 | 2 | 34.8 |
| **psbH** | 222 | 74 | 1 | 0.45 | 1 | 38.3 |
| **cemA** | 690 | 230 | 3 | 0.43 | 0 | 32.9 |
| **rpl22** | 465 | 155 | 2 | 0.43 | 1 | 34.4 |
| **psbC** | 1,422 | 474 | 6 | 0.42 | 3 | 43.1 |
| **petD** | 486 | 162 | 2 | 0.41 | 0 | 39.3 |
| **psaB** | 2,205 | 735 | 9 | 0.41 | 5 | 40.3 |
| **atpB** | 1,497 | 499 | 6 | 0.40 | 5 | 42.2 |
| **ycf3** | 507 | 169 | 2 | 0.39 | 2 | 39.5 |
| **psbB** | 1,527 | 509 | 6 | 0.39 | 3 | 43.4 |
| **psaC** | 257 | 86 | 1 | 0.39 | 1 | 42.3 |
| **rps15** | 279 | 93 | 1 | 0.36 | 1 | 32.2 |
| **clpP** | 591 | 197 | 2 | 0.34 | 2 | 42.3 |
| **petB** | 648 | 216 | 2 | 0.31 | 2 | 39.8 |
| **rps12** | 357 | 119 | 1 | 0.28 | 1 | 44.5 |
| **rpl14** | 369 | 123 | 1 | 0.27 | 1 | 40.4 |
| **psaA** | 2,253 | 751 | 6 | 0.27 | 3 | 43.1 |
| **atpE** | 402 | 134 | 1 | 0.25 | 0 | 39.8 |
| **rps11** | 411 | 137 | 1 | 0.24 | 0 | 45.7 |
| **ycf2** | 6,114 | 2,038 | 14 | 0.23 | 8 | 37.7 |
| **ndhJ** | 477 | 159 | 1 | 0.21 | 1 | 39.6 |
| **rpl2** | 825 | 275 | 1 | 0.12 | 0 | 43.6 |
| **atpF** | 1,260 | 420 | 1 | 0.08 | 0 | 37.3 |
| **ndhB** | 1,533 | 511 | 1 | 0.07 | 0 | 37.2 |
| **atpH** | 246 | 82 | 0 | 0.00 | 0 | 44.7 |
| **infA** | 234 | 78 | 0 | 0.00 | 0 | 35.9 |
| **petL** | 96 | 32 | 0 | 0.00 | 0 | 35.4 |
| **petN** | 90 | 30 | 0 | 0.00 | 0 | 41.1 |
| **psaJ** | 129 | 43 | 0 | 0.00 | 0 | 41.9 |
| **psbE** | 252 | 84 | 0 | 0.00 | 0 | 41.7 |
| **psbI** | 111 | 37 | 0 | 0.00 | 0 | 36.0 |
| **psbK** | 180 | 60 | 0 | 0.00 | 0 | 37.8 |
| **psbL** | 117 | 39 | 0 | 0.00 | 0 | 34.2 |
| **psbM** | 105 | 35 | 0 | 0.00 | 0 | 27.6 |
| **psbN** | 132 | 44 | 0 | 0.00 | 0 | 43.9 |
| **psbZ** | 189 | 63 | 0 | 0.00 | 0 | 36.5 |
| **rpl23** | 282 | 94 | 0 | 0.00 | 0 | 39.4 |
| **rpl36** | 114 | 38 | 0 | 0.00 | 0 | 35.1 |
| **rps7** | 468 | 156 | 0 | 0.00 | 0 | 41.0 |

**Note**: Length (bp): alignment length, Length translated: length of alignment translated in amino acids, Var. sites: number of variable sites, Var. sites %: percentage of variable sites, Pi sites: parsimony informative sites, GC%: percentage of GC content.
