# Supplementary material for: Extremely low nucleotide diversity among thirty-six new chloroplast genome sequences from Aldama (Heliantheae, Asteraceae) and comparative chloroplast genomics analyses with closely related genera
Source: PeerJ. 2021 Feb 24;9:e10886. doi: 10.7717/peerj.10886 (PMC7912680; doi:10.7717/peerj.10886)
Supplement: Supplemental Information 5 [file peerj-09-10886-s005.docx]

**Supplemental Table 3.** Results from the distribution of SSRs in all plastomes of the *Aldama* species studied here and five other Heliantheae genera.

| **Species** | **SSRs total** | **Unit size** | | | | | | **SSR per plastome region** | | | **Repeat type** | | | |
| --- | --- | --- | --- | --- | --- | --- | --- | --- | --- | --- | --- | --- | --- | --- |
|  |  | **mono** | **di** | **tri** | **tetra** | **penta** | **hexa** | **LSC** | **IR** | **SSC** | **A/T repeats** | **% of A/T repeats from total** | **A repeats** | **T repeats** |
| *Aldama canescens* | 50 | 35 | 5 | 5 | 5 | 0 | 0 | 40 | 2 | 8 | 34 | 68.0 | 17 | 17 |
| *Aldama dentata 2* | 57 | 44 | 5 | 4 | 4 | 0 | 0 | 45 | 2 | 10 | 42 | 73.7 | 25 | 17 |
| *Aldama excelsa* | 54 | 38 | 5 | 5 | 5 | 0 | 1 | 42 | 2 | 10 | 37 | 68.5 | 21 | 16 |
| *Aldama filifolia* | 50 | 37 | 5 | 3 | 5 | 0 | 0 | 39 | 2 | 9 | 35 | 70.0 | 20 | 15 |
| *Aldama grandiflora* | 51 | 37 | 5 | 4 | 5 | 0 | 0 | 41 | 2 | 8 | 36 | 70.6 | 22 | 14 |
| *Aldama linearis* | 47 | 34 | 5 | 4 | 4 | 0 | 0 | 37 | 2 | 8 | 33 | 70.2 | 18 | 15 |
| *Aldama macrorhiza* | 50 | 37 | 5 | 3 | 5 | 0 | 0 | 38 | 2 | 10 | 36 | 72.0 | 22 | 14 |
| *Aldama rubra* | 56 | 42 | 5 | 4 | 5 | 0 | 0 | 44 | 3 | 9 | 41 | 73.2 | 25 | 16 |
| *Aldama trichophylla 2* | 49 | 35 | 5 | 4 | 5 | 0 | 0 | 39 | 1 | 9 | 34 | 69.4 | 19 | 15 |
| *Aldama veredensis* | 49 | 35 | 5 | 4 | 5 | 0 | 0 | 40 | 1 | 8 | 33 | 67.3 | 20 | 13 |
| **Outgroups** |  |  |  |  |  |  |  |  |  |  |  |  |  |  |
| *Dimerostemma asperatum* | 38 | 23 | 5 | 6 | 3 | 1 | 0 | 28 | 1 | 9 | 23 | 60.5 | 14 | 9 |
| *Helianthus tuberosus* | 49 | 37 | 5 | 3 | 4 | 0 | 0 | 40 | 1 | 8 | 37 | 75.5 | 21 | 16 |
| *Iostephane heterophylla* | 56 | 41 | 5 | 4 | 5 | 0 | 1 | 44 | 2 | 10 | 40 | 71.4 | 25 | 15 |
| *Pappobolus lanatus* var*. lanatus* | 54 | 39 | 4 | 5 | 5 | 1 | 0 | 45 | 0 | 9 | 37 | 68.5 | 22 | 15 |
| *Tithonia diversifolia* | 50 | 37 | 4 | 5 | 4 | 0 | 0 | 39 | 2 | 9 | 36 | 72.0 | 20 | 16 |
