# Supplementary figures and images for: Extremely low nucleotide diversity among thirty-six new chloroplast genome sequences from Aldama (Heliantheae, Asteraceae) and comparative chloroplast genomics analyses with closely related genera
Source: PeerJ. 2021 Feb 24;9:e10886. doi: 10.7717/peerj.10886 (PMC7912680; doi:10.7717/peerj.10886)

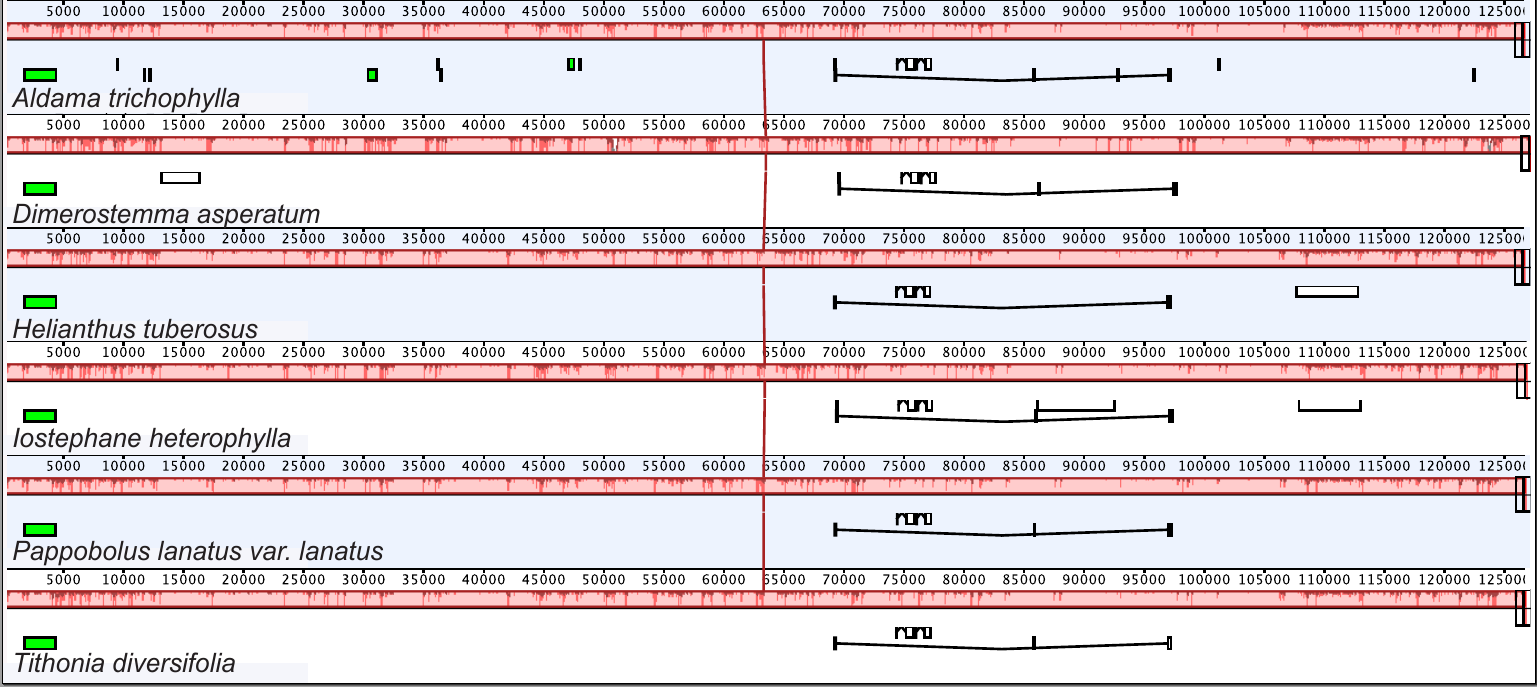

Supplement: Supplemental Information 6 — The red block indicates a single synteny block showing that the plastomes from these six genera are very conserved. [file peerj-09-10886-s006.png]

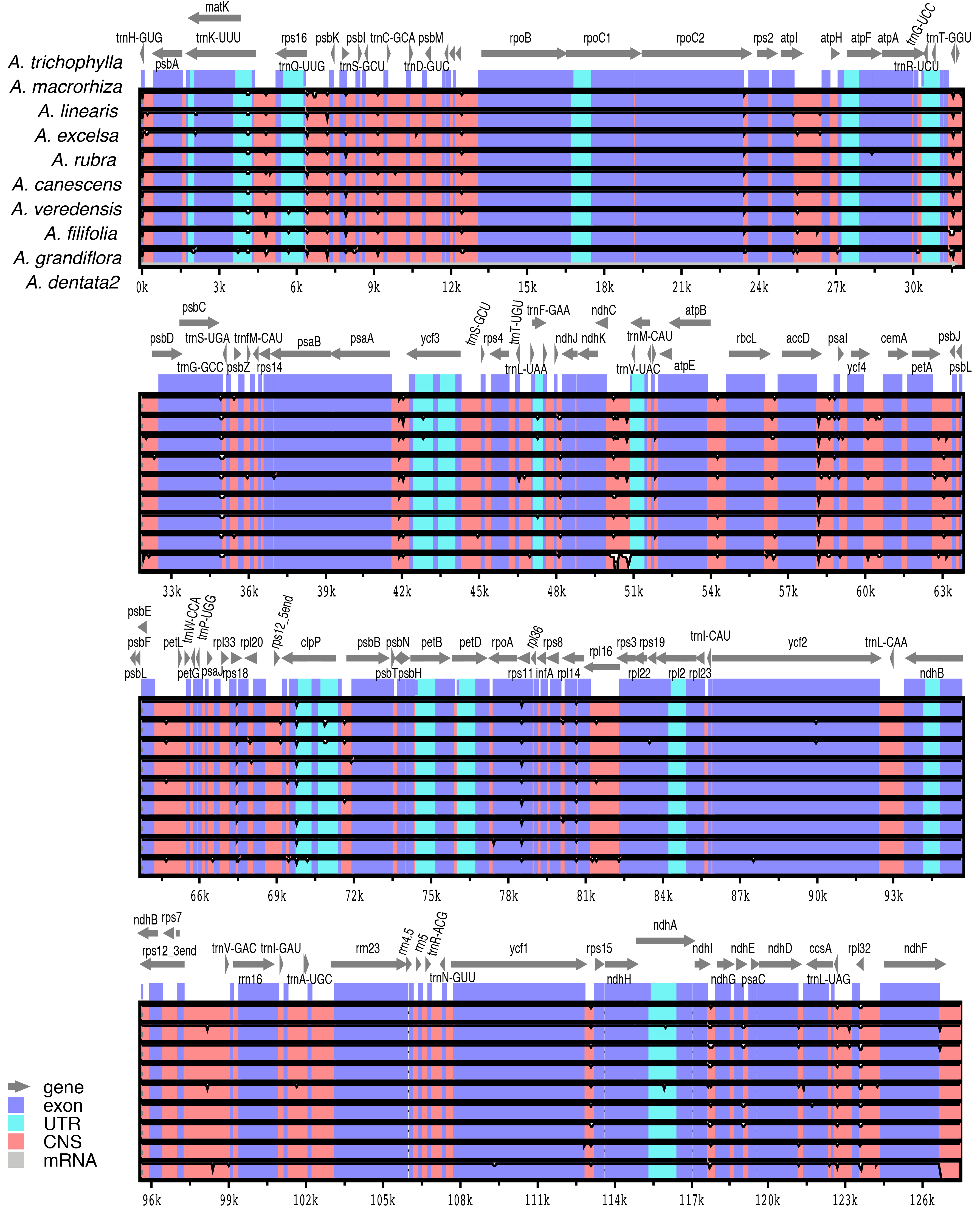

Supplement: Supplemental Information 7 — The plastome of A. trichophylla was used as reference. Dark blue blocks indicate conserved genes (CNS), light blue blocks indicate conserved introns (UTR), and red blocks indicate conserved noncoding sequences (CNS). White blocks represent regions with sequence variation among the plastomes. The vertical axis indicates sequence alignment similarity of 50–100%. [file peerj-09-10886-s007.png]
