# Supplementary material for: Extremely low nucleotide diversity among thirty-six new chloroplast genome sequences from Aldama (Heliantheae, Asteraceae) and comparative chloroplast genomics analyses with closely related genera
Source: PeerJ. 2021 Feb 24;9:e10886. doi: 10.7717/peerj.10886 (PMC7912680; doi:10.7717/peerj.10886)
Supplement: Supplemental Information 10 [file peerj-09-10886-s010.docx]

Statistical Significance Results for run number 1567461694

Log-likelihood for M8 model allowing for positive selection: -2134.61

Log-likelihood for null model M8a which does not allow positive selection: -2137.17

Likelihood ratio test between the two models shows a significance level of: 0.05

Selecton Bayesian Ka/Ks Results

Displayed on sequence 1

==========================================================================================================================================

POS AMINO Ka/Ks [Confidence Interval](* if lower bound > 1) POSTERIOR PROBABILITIES

w = 6.2e-10 4.4e-07 2e-05 0.0003 0.0023 0.013 0.054 0.26 4.7

==========================================================================================================================================

1 M 0.24 [6.2e-10,4.7] 0.12 0.12 0.12 0.12 0.12 0.12 0.12 0.12 0.043

2 S 0.28 [6.2e-10,4.7] 0.12 0.12 0.12 0.12 0.12 0.12 0.12 0.12 0.051

3 P 0.26 [6.2e-10,4.7] 0.12 0.12 0.12 0.12 0.12 0.12 0.12 0.12 0.047

4 Q 0.25 [6.2e-10,4.7] 0.12 0.12 0.12 0.12 0.12 0.12 0.12 0.12 0.044

5 T 0.25 [6.2e-10,4.7] 0.12 0.12 0.12 0.12 0.12 0.12 0.12 0.12 0.045

6 E 0.24 [6.2e-10,4.7] 0.12 0.12 0.12 0.12 0.12 0.12 0.12 0.12 0.042

7 T 0.22 [6.2e-10,4.7] 0.12 0.12 0.12 0.12 0.12 0.12 0.12 0.12 0.037

8 K 0.23 [6.2e-10,4.7] 0.12 0.12 0.12 0.12 0.12 0.12 0.12 0.12 0.039

9 A 0.24 [6.2e-10,4.7] 0.12 0.12 0.12 0.12 0.12 0.12 0.12 0.12 0.042

10 S 0.21 [6.2e-10,4.7] 0.12 0.12 0.12 0.12 0.12 0.12 0.12 0.12 0.035

11 V 0.2 [6.2e-10,4.7] 0.12 0.12 0.12 0.12 0.12 0.12 0.12 0.12 0.034

12 G 0.24 [6.2e-10,4.7] 0.12 0.12 0.12 0.12 0.12 0.12 0.12 0.12 0.043

13 F 0.28 [6.2e-10,4.7] 0.12 0.12 0.12 0.12 0.12 0.12 0.12 0.12 0.05

14 K 0.23 [6.2e-10,4.7] 0.12 0.12 0.12 0.12 0.12 0.12 0.12 0.12 0.039

15 A 0.2 [6.2e-10,4.7] 0.12 0.12 0.12 0.12 0.12 0.12 0.12 0.12 0.034

16 G 0.2 [6.2e-10,4.7] 0.12 0.12 0.12 0.12 0.12 0.12 0.12 0.12 0.034

17 V 0.2 [6.2e-10,4.7] 0.12 0.12 0.12 0.12 0.12 0.12 0.12 0.12 0.034

18 K 0.23 [6.2e-10,4.7] 0.12 0.12 0.12 0.12 0.12 0.12 0.12 0.12 0.039

19 D 0.17 [6.2e-10,4.7] 0.12 0.12 0.12 0.12 0.12 0.12 0.12 0.12 0.029

20 Y 0.22 [6.2e-10,4.7] 0.12 0.12 0.12 0.12 0.12 0.12 0.12 0.12 0.038

21 K 0.23 [6.2e-10,4.7] 0.12 0.12 0.12 0.12 0.12 0.12 0.12 0.12 0.039

22 L 0.29 [6.2e-10,4.7] 0.12 0.12 0.12 0.12 0.12 0.12 0.12 0.12 0.053

23 T 0.22 [6.2e-10,4.7] 0.12 0.12 0.12 0.12 0.12 0.12 0.12 0.12 0.037

24 Y 0.22 [6.2e-10,4.7] 0.12 0.12 0.12 0.12 0.12 0.12 0.12 0.12 0.038

25 Y 0.22 [6.2e-10,4.7] 0.12 0.12 0.12 0.12 0.12 0.12 0.12 0.12 0.038

26 T 0.22 [6.2e-10,4.7] 0.12 0.12 0.12 0.12 0.12 0.12 0.12 0.12 0.037

27 P 0.23 [6.2e-10,4.7] 0.12 0.12 0.12 0.12 0.12 0.12 0.12 0.12 0.041

28 E 0.21 [6.2e-10,4.7] 0.12 0.12 0.12 0.12 0.12 0.12 0.12 0.12 0.036

29 Y 0.22 [6.2e-10,4.7] 0.12 0.12 0.12 0.12 0.12 0.12 0.12 0.12 0.038

30 E 0.21 [6.2e-10,4.7] 0.12 0.12 0.12 0.12 0.12 0.12 0.12 0.12 0.036

31 T 0.28 [6.2e-10,4.7] 0.12 0.12 0.12 0.12 0.12 0.12 0.12 0.12 0.052

32 K 0.26 [6.2e-10,4.7] 0.12 0.12 0.12 0.12 0.12 0.12 0.12 0.12 0.046

33 D 0.17 [6.2e-10,4.7] 0.12 0.12 0.12 0.12 0.12 0.12 0.12 0.12 0.029

34 T 0.22 [6.2e-10,4.7] 0.12 0.12 0.12 0.12 0.12 0.12 0.12 0.12 0.037

35 D 0.17 [6.2e-10,4.7] 0.12 0.12 0.12 0.12 0.12 0.12 0.12 0.12 0.029

36 I 0.28 [6.2e-10,4.7] 0.12 0.12 0.12 0.12 0.12 0.12 0.12 0.12 0.05

37 L 0.29 [6.2e-10,4.7] 0.12 0.12 0.12 0.12 0.12 0.12 0.12 0.12 0.053

38 A 0.24 [6.2e-10,4.7] 0.12 0.12 0.12 0.12 0.12 0.12 0.12 0.12 0.042

39 A 0.24 [6.2e-10,4.7] 0.12 0.12 0.12 0.12 0.12 0.12 0.12 0.12 0.042

40 F 0.22 [6.2e-10,4.7] 0.12 0.12 0.12 0.12 0.12 0.12 0.12 0.12 0.038

41 R 0.28 [6.2e-10,4.7] 0.12 0.12 0.12 0.12 0.12 0.12 0.12 0.12 0.052

42 V 0.24 [6.2e-10,4.7] 0.12 0.12 0.12 0.12 0.12 0.12 0.12 0.12 0.042

43 T 0.22 [6.2e-10,4.7] 0.12 0.12 0.12 0.12 0.12 0.12 0.12 0.12 0.037

44 P 0.23 [6.2e-10,4.7] 0.12 0.12 0.12 0.12 0.12 0.12 0.12 0.12 0.041

45 Q 0.25 [6.2e-10,4.7] 0.12 0.12 0.12 0.12 0.12 0.12 0.12 0.12 0.044

46 P 0.23 [6.2e-10,4.7] 0.12 0.12 0.12 0.12 0.12 0.12 0.12 0.12 0.041

47 G 0.24 [6.2e-10,4.7] 0.12 0.12 0.12 0.12 0.12 0.12 0.12 0.12 0.043

48 V 0.2 [6.2e-10,4.7] 0.12 0.12 0.12 0.12 0.12 0.12 0.12 0.12 0.034

49 P 0.29 [6.2e-10,4.7] 0.12 0.12 0.12 0.12 0.12 0.12 0.12 0.12 0.054

50 P 0.23 [6.2e-10,4.7] 0.12 0.12 0.12 0.12 0.12 0.12 0.12 0.12 0.041

51 E 0.21 [6.2e-10,4.7] 0.12 0.12 0.12 0.12 0.12 0.12 0.12 0.12 0.036

52 E 0.21 [6.2e-10,4.7] 0.12 0.12 0.12 0.12 0.12 0.12 0.12 0.12 0.036

53 A 0.24 [6.2e-10,4.7] 0.12 0.12 0.12 0.12 0.12 0.12 0.12 0.12 0.042

54 G 0.28 [6.2e-10,4.7] 0.12 0.12 0.12 0.12 0.12 0.12 0.12 0.12 0.051

55 A 0.28 [6.2e-10,4.7] 0.12 0.12 0.12 0.12 0.12 0.12 0.12 0.12 0.05

56 A 0.24 [6.2e-10,4.7] 0.12 0.12 0.12 0.12 0.12 0.12 0.12 0.12 0.042

57 V 0.24 [6.2e-10,4.7] 0.12 0.12 0.12 0.12 0.12 0.12 0.12 0.12 0.042

58 A 0.2 [6.2e-10,4.7] 0.12 0.12 0.12 0.12 0.12 0.12 0.12 0.12 0.034

59 A 0.28 [6.2e-10,4.7] 0.12 0.12 0.12 0.12 0.12 0.12 0.12 0.12 0.05

60 E 0.21 [6.2e-10,4.7] 0.12 0.12 0.12 0.12 0.12 0.12 0.12 0.12 0.036

61 S 0.23 [6.2e-10,4.7] 0.12 0.12 0.12 0.12 0.12 0.12 0.12 0.12 0.041

62 S 0.23 [6.2e-10,4.7] 0.12 0.12 0.12 0.12 0.12 0.12 0.12 0.12 0.041

63 T 0.22 [6.2e-10,4.7] 0.12 0.12 0.12 0.12 0.12 0.12 0.12 0.12 0.037

64 G 0.2 [6.2e-10,4.7] 0.12 0.12 0.12 0.12 0.12 0.12 0.12 0.12 0.034

65 T 0.25 [6.2e-10,4.7] 0.12 0.12 0.12 0.12 0.12 0.12 0.12 0.12 0.045

66 W 0.29 [6.2e-10,4.7] 0.12 0.12 0.12 0.12 0.12 0.12 0.12 0.12 0.053

67 T 0.25 [6.2e-10,4.7] 0.12 0.12 0.12 0.12 0.12 0.12 0.12 0.12 0.045

68 T 0.22 [6.2e-10,4.7] 0.12 0.12 0.12 0.12 0.12 0.12 0.12 0.12 0.037

69 V 0.24 [6.2e-10,4.7] 0.12 0.12 0.12 0.12 0.12 0.12 0.12 0.12 0.042

70 W 0.29 [6.2e-10,4.7] 0.12 0.12 0.12 0.12 0.12 0.12 0.12 0.12 0.053

71 T 0.28 [6.2e-10,4.7] 0.12 0.12 0.12 0.12 0.12 0.12 0.12 0.12 0.052

72 D 0.17 [6.2e-10,4.7] 0.12 0.12 0.12 0.12 0.12 0.12 0.12 0.12 0.029

73 G 0.24 [6.2e-10,4.7] 0.12 0.12 0.12 0.12 0.12 0.12 0.12 0.12 0.043

74 L 0.23 [6.2e-10,4.7] 0.12 0.12 0.12 0.12 0.12 0.12 0.12 0.12 0.04

75 T 0.28 [6.2e-10,4.7] 0.12 0.12 0.12 0.12 0.12 0.12 0.12 0.12 0.052

76 S 0.27 [6.2e-10,4.7] 0.12 0.12 0.12 0.12 0.12 0.12 0.12 0.12 0.049

77 L 0.23 [6.2e-10,4.7] 0.12 0.12 0.12 0.12 0.12 0.12 0.12 0.12 0.04

78 D 0.24 [6.2e-10,4.7] 0.12 0.12 0.12 0.12 0.12 0.12 0.12 0.12 0.043

79 R 0.23 [6.2e-10,4.7] 0.12 0.12 0.12 0.12 0.12 0.12 0.12 0.12 0.041

80 Y 0.28 [6.2e-10,4.7] 0.12 0.12 0.12 0.12 0.12 0.12 0.12 0.12 0.052

81 K 0.23 [6.2e-10,4.7] 0.12 0.12 0.12 0.12 0.12 0.12 0.12 0.12 0.039

82 G 0.28 [6.2e-10,4.7] 0.12 0.12 0.12 0.12 0.12 0.12 0.12 0.12 0.05

83 R 0.28 [6.2e-10,4.7] 0.12 0.12 0.12 0.12 0.12 0.12 0.12 0.12 0.052

84 C 0.29 [6.2e-10,4.7] 0.12 0.12 0.12 0.12 0.12 0.12 0.12 0.12 0.053

85 Y 0.22 [6.2e-10,4.7] 0.12 0.12 0.12 0.12 0.12 0.12 0.12 0.12 0.038

86 G 0.24 [6.2e-10,4.7] 0.12 0.12 0.12 0.12 0.12 0.12 0.12 0.12 0.043

87 L 4 [0.054,4.7] 3.2e-10 2.3e-07 1.1e-05 0.00015 0.0012 0.0065 0.028 0.13 0.83

88 E 0.24 [6.2e-10,4.7] 0.12 0.12 0.12 0.12 0.12 0.12 0.12 0.12 0.042

89 P 0.23 [6.2e-10,4.7] 0.12 0.12 0.12 0.12 0.12 0.12 0.12 0.12 0.041

90 V 0.2 [6.2e-10,4.7] 0.12 0.12 0.12 0.12 0.12 0.12 0.12 0.12 0.034

91 P 4.7 [4.7,4.7]* 5.1e-20 2.6e-14 5.4e-11 1.2e-08 7.1e-07 2e-05 0.00038 0.0089 0.99

92 G 0.24 [6.2e-10,4.7] 0.12 0.12 0.12 0.12 0.12 0.12 0.12 0.12 0.043

93 E 0.21 [6.2e-10,4.7] 0.12 0.12 0.12 0.12 0.12 0.12 0.12 0.12 0.036

94 E 4 [0.054,4.7] 3.1e-10 2.2e-07 1e-05 0.00015 0.0012 0.0062 0.027 0.13 0.84

95 N 0.19 [6.2e-10,4.7] 0.12 0.12 0.12 0.12 0.12 0.12 0.12 0.12 0.032

96 Q 0.25 [6.2e-10,4.7] 0.12 0.12 0.12 0.12 0.12 0.12 0.12 0.12 0.044

97 Y 0.22 [6.2e-10,4.7] 0.12 0.12 0.12 0.12 0.12 0.12 0.12 0.12 0.038

98 I 0.21 [6.2e-10,4.7] 0.12 0.12 0.12 0.12 0.12 0.12 0.12 0.12 0.036

99 A 0.2 [6.2e-10,4.7] 0.12 0.12 0.12 0.12 0.12 0.12 0.12 0.12 0.034

100 Y 0.22 [6.2e-10,4.7] 0.12 0.12 0.12 0.12 0.12 0.12 0.12 0.12 0.038

101 V 0.24 [6.2e-10,4.7] 0.12 0.12 0.12 0.12 0.12 0.12 0.12 0.12 0.042

102 A 0.2 [6.2e-10,4.7] 0.12 0.12 0.12 0.12 0.12 0.12 0.12 0.12 0.034

103 Y 0.29 [6.2e-10,4.7] 0.12 0.12 0.12 0.12 0.12 0.12 0.12 0.12 0.052

104 P 0.26 [6.2e-10,4.7] 0.12 0.12 0.12 0.12 0.12 0.12 0.12 0.12 0.047

105 L 0.27 [6.2e-10,4.7] 0.12 0.12 0.12 0.12 0.12 0.12 0.12 0.12 0.049

106 D 0.24 [6.2e-10,4.7] 0.12 0.12 0.12 0.12 0.12 0.12 0.12 0.12 0.043

107 L 0.23 [6.2e-10,4.7] 0.12 0.12 0.12 0.12 0.12 0.12 0.12 0.12 0.04

108 F 0.22 [6.2e-10,4.7] 0.12 0.12 0.12 0.12 0.12 0.12 0.12 0.12 0.038

109 E 0.21 [6.2e-10,4.7] 0.12 0.12 0.12 0.12 0.12 0.12 0.12 0.12 0.036

110 E 0.21 [6.2e-10,4.7] 0.12 0.12 0.12 0.12 0.12 0.12 0.12 0.12 0.036

111 G 0.2 [6.2e-10,4.7] 0.12 0.12 0.12 0.12 0.12 0.12 0.12 0.12 0.034

112 S 0.23 [6.2e-10,4.7] 0.12 0.12 0.12 0.12 0.12 0.12 0.12 0.12 0.041

113 V 0.2 [6.2e-10,4.7] 0.12 0.12 0.12 0.12 0.12 0.12 0.12 0.12 0.034

114 T 0.22 [6.2e-10,4.7] 0.12 0.12 0.12 0.12 0.12 0.12 0.12 0.12 0.037

115 N 0.26 [6.2e-10,4.7] 0.12 0.12 0.12 0.12 0.12 0.12 0.12 0.12 0.046

116 M 0.24 [6.2e-10,4.7] 0.12 0.12 0.12 0.12 0.12 0.12 0.12 0.12 0.043

117 F 0.22 [6.2e-10,4.7] 0.12 0.12 0.12 0.12 0.12 0.12 0.12 0.12 0.038

118 T 0.22 [6.2e-10,4.7] 0.12 0.12 0.12 0.12 0.12 0.12 0.12 0.12 0.037

119 S 0.29 [6.2e-10,4.7] 0.12 0.12 0.12 0.12 0.12 0.12 0.12 0.12 0.054

120 I 0.21 [6.2e-10,4.7] 0.12 0.12 0.12 0.12 0.12 0.12 0.12 0.12 0.036

121 V 0.24 [6.2e-10,4.7] 0.12 0.12 0.12 0.12 0.12 0.12 0.12 0.12 0.042

122 G 0.2 [6.2e-10,4.7] 0.12 0.12 0.12 0.12 0.12 0.12 0.12 0.12 0.034

123 N 0.19 [6.2e-10,4.7] 0.12 0.12 0.12 0.12 0.12 0.12 0.12 0.12 0.032

124 V 0.24 [6.2e-10,4.7] 0.12 0.12 0.12 0.12 0.12 0.12 0.12 0.12 0.042

125 F 0.22 [6.2e-10,4.7] 0.12 0.12 0.12 0.12 0.12 0.12 0.12 0.12 0.038

126 G 0.28 [6.2e-10,4.7] 0.12 0.12 0.12 0.12 0.12 0.12 0.12 0.12 0.051

127 F 0.28 [6.2e-10,4.7] 0.12 0.12 0.12 0.12 0.12 0.12 0.12 0.12 0.05

128 K 0.23 [6.2e-10,4.7] 0.12 0.12 0.12 0.12 0.12 0.12 0.12 0.12 0.039

129 A 0.28 [6.2e-10,4.7] 0.12 0.12 0.12 0.12 0.12 0.12 0.12 0.12 0.05

130 L 0.3 [6.2e-10,4.7] 0.12 0.12 0.12 0.12 0.12 0.12 0.12 0.12 0.055

131 R 0.23 [6.2e-10,4.7] 0.12 0.12 0.12 0.12 0.12 0.12 0.12 0.12 0.041

132 A 0.2 [6.2e-10,4.7] 0.12 0.12 0.12 0.12 0.12 0.12 0.12 0.12 0.034

133 L 0.27 [6.2e-10,4.7] 0.12 0.12 0.12 0.12 0.12 0.12 0.12 0.12 0.049

134 R 0.23 [6.2e-10,4.7] 0.12 0.12 0.12 0.12 0.12 0.12 0.12 0.12 0.041

135 L 0.3 [6.2e-10,4.7] 0.12 0.12 0.12 0.12 0.12 0.12 0.12 0.12 0.055

136 E 0.21 [6.2e-10,4.7] 0.12 0.12 0.12 0.12 0.12 0.12 0.12 0.12 0.036

137 D 0.17 [6.2e-10,4.7] 0.12 0.12 0.12 0.12 0.12 0.12 0.12 0.12 0.029

138 L 0.29 [6.2e-10,4.7] 0.12 0.12 0.12 0.12 0.12 0.12 0.12 0.12 0.053

139 R 0.28 [6.2e-10,4.7] 0.12 0.12 0.12 0.12 0.12 0.12 0.12 0.12 0.052

140 I 0.28 [6.2e-10,4.7] 0.12 0.12 0.12 0.12 0.12 0.12 0.12 0.12 0.05

141 P 0.29 [6.2e-10,4.7] 0.12 0.12 0.12 0.12 0.12 0.12 0.12 0.12 0.054

142 T 0.22 [6.2e-10,4.7] 0.12 0.12 0.12 0.12 0.12 0.12 0.12 0.12 0.037

143 A 0.28 [6.2e-10,4.7] 0.12 0.12 0.12 0.12 0.12 0.12 0.12 0.12 0.051

144 Y 0.22 [6.2e-10,4.7] 0.12 0.12 0.12 0.12 0.12 0.12 0.12 0.12 0.038

145 V 0.2 [6.2e-10,4.7] 0.12 0.12 0.12 0.12 0.12 0.12 0.12 0.12 0.034

146 K 0.23 [6.2e-10,4.7] 0.12 0.12 0.12 0.12 0.12 0.12 0.12 0.12 0.039

147 T 0.22 [6.2e-10,4.7] 0.12 0.12 0.12 0.12 0.12 0.12 0.12 0.12 0.037

148 F 0.28 [6.2e-10,4.7] 0.12 0.12 0.12 0.12 0.12 0.12 0.12 0.12 0.05

149 D 3.9 [0.054,4.7] 3.7e-10 2.6e-07 1.2e-05 0.00018 0.0014 0.0074 0.032 0.15 0.81

150 G 0.2 [6.2e-10,4.7] 0.12 0.12 0.12 0.12 0.12 0.12 0.12 0.12 0.034

151 P 0.29 [6.2e-10,4.7] 0.12 0.12 0.12 0.12 0.12 0.12 0.12 0.12 0.054

152 P 0.23 [6.2e-10,4.7] 0.12 0.12 0.12 0.12 0.12 0.12 0.12 0.12 0.041

153 H 0.27 [6.2e-10,4.7] 0.12 0.12 0.12 0.12 0.12 0.12 0.12 0.12 0.049

154 G 0.2 [6.2e-10,4.7] 0.12 0.12 0.12 0.12 0.12 0.12 0.12 0.12 0.034

155 I 0.28 [6.2e-10,4.7] 0.12 0.12 0.12 0.12 0.12 0.12 0.12 0.12 0.05

156 Q 0.25 [6.2e-10,4.7] 0.12 0.12 0.12 0.12 0.12 0.12 0.12 0.12 0.044

157 V 0.2 [6.2e-10,4.7] 0.12 0.12 0.12 0.12 0.12 0.12 0.12 0.12 0.034

158 E 0.21 [6.2e-10,4.7] 0.12 0.12 0.12 0.12 0.12 0.12 0.12 0.12 0.036

159 R 0.25 [6.2e-10,4.7] 0.12 0.12 0.12 0.12 0.12 0.12 0.12 0.12 0.044

160 D 0.17 [6.2e-10,4.7] 0.12 0.12 0.12 0.12 0.12 0.12 0.12 0.12 0.029

161 K 0.23 [6.2e-10,4.7] 0.12 0.12 0.12 0.12 0.12 0.12 0.12 0.12 0.039

162 L 0.29 [6.2e-10,4.7] 0.12 0.12 0.12 0.12 0.12 0.12 0.12 0.12 0.053

163 N 0.26 [6.2e-10,4.7] 0.12 0.12 0.12 0.12 0.12 0.12 0.12 0.12 0.046

164 K 0.26 [6.2e-10,4.7] 0.12 0.12 0.12 0.12 0.12 0.12 0.12 0.12 0.046

165 Y 0.22 [6.2e-10,4.7] 0.12 0.12 0.12 0.12 0.12 0.12 0.12 0.12 0.038

166 G 0.2 [6.2e-10,4.7] 0.12 0.12 0.12 0.12 0.12 0.12 0.12 0.12 0.034

167 R 0.23 [6.2e-10,4.7] 0.12 0.12 0.12 0.12 0.12 0.12 0.12 0.12 0.041

168 P 0.29 [6.2e-10,4.7] 0.12 0.12 0.12 0.12 0.12 0.12 0.12 0.12 0.054

169 L 0.3 [6.2e-10,4.7] 0.12 0.12 0.12 0.12 0.12 0.12 0.12 0.12 0.055

170 L 0.29 [6.2e-10,4.7] 0.12 0.12 0.12 0.12 0.12 0.12 0.12 0.12 0.053

171 G 0.24 [6.2e-10,4.7] 0.12 0.12 0.12 0.12 0.12 0.12 0.12 0.12 0.043

172 C 0.23 [6.2e-10,4.7] 0.12 0.12 0.12 0.12 0.12 0.12 0.12 0.12 0.041

173 T 0.22 [6.2e-10,4.7] 0.12 0.12 0.12 0.12 0.12 0.12 0.12 0.12 0.037

174 I 0.21 [6.2e-10,4.7] 0.12 0.12 0.12 0.12 0.12 0.12 0.12 0.12 0.036

175 K 0.23 [6.2e-10,4.7] 0.12 0.12 0.12 0.12 0.12 0.12 0.12 0.12 0.039

176 P 0.29 [6.2e-10,4.7] 0.12 0.12 0.12 0.12 0.12 0.12 0.12 0.12 0.054

177 K 0.23 [6.2e-10,4.7] 0.12 0.12 0.12 0.12 0.12 0.12 0.12 0.12 0.039

178 L 0.29 [6.2e-10,4.7] 0.12 0.12 0.12 0.12 0.12 0.12 0.12 0.12 0.053

179 G 0.28 [6.2e-10,4.7] 0.12 0.12 0.12 0.12 0.12 0.12 0.12 0.12 0.051

180 L 0.27 [6.2e-10,4.7] 0.12 0.12 0.12 0.12 0.12 0.12 0.12 0.12 0.049

181 S 0.29 [6.2e-10,4.7] 0.12 0.12 0.12 0.12 0.12 0.12 0.12 0.12 0.054

182 A 0.2 [6.2e-10,4.7] 0.12 0.12 0.12 0.12 0.12 0.12 0.12 0.12 0.034

183 K 0.23 [6.2e-10,4.7] 0.12 0.12 0.12 0.12 0.12 0.12 0.12 0.12 0.039

184 N 0.26 [6.2e-10,4.7] 0.12 0.12 0.12 0.12 0.12 0.12 0.12 0.12 0.046

185 Y 0.29 [6.2e-10,4.7] 0.12 0.12 0.12 0.12 0.12 0.12 0.12 0.12 0.052

186 G 0.2 [6.2e-10,4.7] 0.12 0.12 0.12 0.12 0.12 0.12 0.12 0.12 0.034

187 R 0.25 [6.2e-10,4.7] 0.12 0.12 0.12 0.12 0.12 0.12 0.12 0.12 0.044

188 A 0.2 [6.2e-10,4.7] 0.12 0.12 0.12 0.12 0.12 0.12 0.12 0.12 0.034

189 C 0.23 [6.2e-10,4.7] 0.12 0.12 0.12 0.12 0.12 0.12 0.12 0.12 0.041

190 Y 0.22 [6.2e-10,4.7] 0.12 0.12 0.12 0.12 0.12 0.12 0.12 0.12 0.038

191 E 0.21 [6.2e-10,4.7] 0.12 0.12 0.12 0.12 0.12 0.12 0.12 0.12 0.036

192 C 0.23 [6.2e-10,4.7] 0.12 0.12 0.12 0.12 0.12 0.12 0.12 0.12 0.041

193 L 0.23 [6.2e-10,4.7] 0.12 0.12 0.12 0.12 0.12 0.12 0.12 0.12 0.04

194 R 0.23 [6.2e-10,4.7] 0.12 0.12 0.12 0.12 0.12 0.12 0.12 0.12 0.041

195 G 0.2 [6.2e-10,4.7] 0.12 0.12 0.12 0.12 0.12 0.12 0.12 0.12 0.034

196 G 0.28 [6.2e-10,4.7] 0.12 0.12 0.12 0.12 0.12 0.12 0.12 0.12 0.05

197 L 0.23 [6.2e-10,4.7] 0.12 0.12 0.12 0.12 0.12 0.12 0.12 0.12 0.04

198 D 0.17 [6.2e-10,4.7] 0.12 0.12 0.12 0.12 0.12 0.12 0.12 0.12 0.029

199 F 0.22 [6.2e-10,4.7] 0.12 0.12 0.12 0.12 0.12 0.12 0.12 0.12 0.038

200 T 0.22 [6.2e-10,4.7] 0.12 0.12 0.12 0.12 0.12 0.12 0.12 0.12 0.037

201 K 0.23 [6.2e-10,4.7] 0.12 0.12 0.12 0.12 0.12 0.12 0.12 0.12 0.039

202 D 0.17 [6.2e-10,4.7] 0.12 0.12 0.12 0.12 0.12 0.12 0.12 0.12 0.029

203 D 0.17 [6.2e-10,4.7] 0.12 0.12 0.12 0.12 0.12 0.12 0.12 0.12 0.029

204 E 0.24 [6.2e-10,4.7] 0.12 0.12 0.12 0.12 0.12 0.12 0.12 0.12 0.042

205 N 0.26 [6.2e-10,4.7] 0.12 0.12 0.12 0.12 0.12 0.12 0.12 0.12 0.046

206 V 0.28 [6.2e-10,4.7] 0.12 0.12 0.12 0.12 0.12 0.12 0.12 0.12 0.051

207 N 0.26 [6.2e-10,4.7] 0.12 0.12 0.12 0.12 0.12 0.12 0.12 0.12 0.046

208 S 0.29 [6.2e-10,4.7] 0.12 0.12 0.12 0.12 0.12 0.12 0.12 0.12 0.054

209 Q 0.25 [6.2e-10,4.7] 0.12 0.12 0.12 0.12 0.12 0.12 0.12 0.12 0.044

210 P 0.26 [6.2e-10,4.7] 0.12 0.12 0.12 0.12 0.12 0.12 0.12 0.12 0.047

211 F 0.22 [6.2e-10,4.7] 0.12 0.12 0.12 0.12 0.12 0.12 0.12 0.12 0.038

212 M 0.24 [6.2e-10,4.7] 0.12 0.12 0.12 0.12 0.12 0.12 0.12 0.12 0.043

213 R 0.23 [6.2e-10,4.7] 0.12 0.12 0.12 0.12 0.12 0.12 0.12 0.12 0.041

214 W 0.29 [6.2e-10,4.7] 0.12 0.12 0.12 0.12 0.12 0.12 0.12 0.12 0.053

215 R 0.25 [6.2e-10,4.7] 0.12 0.12 0.12 0.12 0.12 0.12 0.12 0.12 0.044

216 D 0.24 [6.2e-10,4.7] 0.12 0.12 0.12 0.12 0.12 0.12 0.12 0.12 0.043

217 R 0.23 [6.2e-10,4.7] 0.12 0.12 0.12 0.12 0.12 0.12 0.12 0.12 0.041

218 F 0.28 [6.2e-10,4.7] 0.12 0.12 0.12 0.12 0.12 0.12 0.12 0.12 0.05

219 L 0.27 [6.2e-10,4.7] 0.12 0.12 0.12 0.12 0.12 0.12 0.12 0.12 0.049

220 F 0.22 [6.2e-10,4.7] 0.12 0.12 0.12 0.12 0.12 0.12 0.12 0.12 0.038

221 C 0.23 [6.2e-10,4.7] 0.12 0.12 0.12 0.12 0.12 0.12 0.12 0.12 0.041

222 A 0.28 [6.2e-10,4.7] 0.12 0.12 0.12 0.12 0.12 0.12 0.12 0.12 0.05

223 E 0.21 [6.2e-10,4.7] 0.12 0.12 0.12 0.12 0.12 0.12 0.12 0.12 0.036

224 A 0.2 [6.2e-10,4.7] 0.12 0.12 0.12 0.12 0.12 0.12 0.12 0.12 0.034

225 I 0.21 [6.2e-10,4.7] 0.12 0.12 0.12 0.12 0.12 0.12 0.12 0.12 0.036

226 Y 0.22 [6.2e-10,4.7] 0.12 0.12 0.12 0.12 0.12 0.12 0.12 0.12 0.038

227 K 0.23 [6.2e-10,4.7] 0.12 0.12 0.12 0.12 0.12 0.12 0.12 0.12 0.039

228 A 0.24 [6.2e-10,4.7] 0.12 0.12 0.12 0.12 0.12 0.12 0.12 0.12 0.042

229 Q 0.25 [6.2e-10,4.7] 0.12 0.12 0.12 0.12 0.12 0.12 0.12 0.12 0.044

230 A 0.2 [6.2e-10,4.7] 0.12 0.12 0.12 0.12 0.12 0.12 0.12 0.12 0.034

231 E 0.21 [6.2e-10,4.7] 0.12 0.12 0.12 0.12 0.12 0.12 0.12 0.12 0.036

232 T 0.25 [6.2e-10,4.7] 0.12 0.12 0.12 0.12 0.12 0.12 0.12 0.12 0.045

233 G 0.2 [6.2e-10,4.7] 0.12 0.12 0.12 0.12 0.12 0.12 0.12 0.12 0.034

234 E 0.21 [6.2e-10,4.7] 0.12 0.12 0.12 0.12 0.12 0.12 0.12 0.12 0.036

235 I 0.28 [6.2e-10,4.7] 0.12 0.12 0.12 0.12 0.12 0.12 0.12 0.12 0.05

236 K 0.23 [6.2e-10,4.7] 0.12 0.12 0.12 0.12 0.12 0.12 0.12 0.12 0.039

237 G 0.28 [6.2e-10,4.7] 0.12 0.12 0.12 0.12 0.12 0.12 0.12 0.12 0.051

238 H 0.21 [6.2e-10,4.7] 0.12 0.12 0.12 0.12 0.12 0.12 0.12 0.12 0.035

239 Y 0.29 [6.2e-10,4.7] 0.12 0.12 0.12 0.12 0.12 0.12 0.12 0.12 0.052

240 L 0.29 [6.2e-10,4.7] 0.12 0.12 0.12 0.12 0.12 0.12 0.12 0.12 0.053

241 N 0.19 [6.2e-10,4.7] 0.12 0.12 0.12 0.12 0.12 0.12 0.12 0.12 0.032

242 A 0.2 [6.2e-10,4.7] 0.12 0.12 0.12 0.12 0.12 0.12 0.12 0.12 0.034

243 T 0.22 [6.2e-10,4.7] 0.12 0.12 0.12 0.12 0.12 0.12 0.12 0.12 0.037

244 A 0.28 [6.2e-10,4.7] 0.12 0.12 0.12 0.12 0.12 0.12 0.12 0.12 0.051

245 G 0.2 [6.2e-10,4.7] 0.12 0.12 0.12 0.12 0.12 0.12 0.12 0.12 0.034

246 N 0.19 [6.2e-10,4.7] 0.12 0.12 0.12 0.12 0.12 0.12 0.12 0.12 0.032

247 C 4.7 [4.7,4.7]* 4e-36 4.4e-12 7.2e-11 8.8e-09 5.4e-07 1.6e-05 0.00029 0.0069 0.99

248 E 0.21 [6.2e-10,4.7] 0.12 0.12 0.12 0.12 0.12 0.12 0.12 0.12 0.036

249 D 4 [0.054,4.7] 3.2e-10 2.3e-07 1.1e-05 0.00015 0.0012 0.0065 0.028 0.13 0.83

250 M 0.24 [6.2e-10,4.7] 0.12 0.12 0.12 0.12 0.12 0.12 0.12 0.12 0.043

251 M 4 [0.054,4.7] 3.1e-10 2.2e-07 1e-05 0.00015 0.0012 0.0062 0.027 0.13 0.84

252 K 0.23 [6.2e-10,4.7] 0.12 0.12 0.12 0.12 0.12 0.12 0.12 0.12 0.039

253 R 0.27 [6.2e-10,4.7] 0.12 0.12 0.12 0.12 0.12 0.12 0.12 0.12 0.049

254 A 0.2 [6.2e-10,4.7] 0.12 0.12 0.12 0.12 0.12 0.12 0.12 0.12 0.034

255 V 4 [0.054,4.7] 3.1e-10 2.2e-07 1e-05 0.00015 0.0012 0.0062 0.027 0.13 0.84

256 F 0.22 [6.2e-10,4.7] 0.12 0.12 0.12 0.12 0.12 0.12 0.12 0.12 0.038

257 A 0.28 [6.2e-10,4.7] 0.12 0.12 0.12 0.12 0.12 0.12 0.12 0.12 0.05

258 R 0.25 [6.2e-10,4.7] 0.12 0.12 0.12 0.12 0.12 0.12 0.12 0.12 0.044

259 E 0.21 [6.2e-10,4.7] 0.12 0.12 0.12 0.12 0.12 0.12 0.12 0.12 0.036

260 L 0.29 [6.2e-10,4.7] 0.12 0.12 0.12 0.12 0.12 0.12 0.12 0.12 0.053

261 G 0.24 [6.2e-10,4.7] 0.12 0.12 0.12 0.12 0.12 0.12 0.12 0.12 0.043

262 V 0.2 [6.2e-10,4.7] 0.12 0.12 0.12 0.12 0.12 0.12 0.12 0.12 0.034

263 P 0.23 [6.2e-10,4.7] 0.12 0.12 0.12 0.12 0.12 0.12 0.12 0.12 0.041

264 I 0.28 [6.2e-10,4.7] 0.12 0.12 0.12 0.12 0.12 0.12 0.12 0.12 0.05

265 V 0.24 [6.2e-10,4.7] 0.12 0.12 0.12 0.12 0.12 0.12 0.12 0.12 0.042

266 M 0.24 [6.2e-10,4.7] 0.12 0.12 0.12 0.12 0.12 0.12 0.12 0.12 0.043

267 H 0.21 [6.2e-10,4.7] 0.12 0.12 0.12 0.12 0.12 0.12 0.12 0.12 0.035

268 D 0.24 [6.2e-10,4.7] 0.12 0.12 0.12 0.12 0.12 0.12 0.12 0.12 0.043

269 Y 0.29 [6.2e-10,4.7] 0.12 0.12 0.12 0.12 0.12 0.12 0.12 0.12 0.052

270 L 0.27 [6.2e-10,4.7] 0.12 0.12 0.12 0.12 0.12 0.12 0.12 0.12 0.049

271 T 0.25 [6.2e-10,4.7] 0.12 0.12 0.12 0.12 0.12 0.12 0.12 0.12 0.045

272 G 0.2 [6.2e-10,4.7] 0.12 0.12 0.12 0.12 0.12 0.12 0.12 0.12 0.034

273 G 0.24 [6.2e-10,4.7] 0.12 0.12 0.12 0.12 0.12 0.12 0.12 0.12 0.043

274 F 0.28 [6.2e-10,4.7] 0.12 0.12 0.12 0.12 0.12 0.12 0.12 0.12 0.05

275 T 0.22 [6.2e-10,4.7] 0.12 0.12 0.12 0.12 0.12 0.12 0.12 0.12 0.037

276 A 0.24 [6.2e-10,4.7] 0.12 0.12 0.12 0.12 0.12 0.12 0.12 0.12 0.042

277 N 0.19 [6.2e-10,4.7] 0.12 0.12 0.12 0.12 0.12 0.12 0.12 0.12 0.032

278 T 0.22 [6.2e-10,4.7] 0.12 0.12 0.12 0.12 0.12 0.12 0.12 0.12 0.037

279 S 0.27 [6.2e-10,4.7] 0.12 0.12 0.12 0.12 0.12 0.12 0.12 0.12 0.049

280 L 0.29 [6.2e-10,4.7] 0.12 0.12 0.12 0.12 0.12 0.12 0.12 0.12 0.053

281 A 3.9 [0.054,4.7] 3.4e-10 2.4e-07 1.1e-05 0.00016 0.0013 0.0068 0.029 0.14 0.82

282 H 0.21 [6.2e-10,4.7] 0.12 0.12 0.12 0.12 0.12 0.12 0.12 0.12 0.035

283 Y 0.22 [6.2e-10,4.7] 0.12 0.12 0.12 0.12 0.12 0.12 0.12 0.12 0.038

284 C 0.29 [6.2e-10,4.7] 0.12 0.12 0.12 0.12 0.12 0.12 0.12 0.12 0.053

285 R 0.28 [6.2e-10,4.7] 0.12 0.12 0.12 0.12 0.12 0.12 0.12 0.12 0.052

286 D 0.23 [6.2e-10,4.7] 0.12 0.12 0.12 0.12 0.12 0.12 0.12 0.12 0.041

287 N 0.19 [6.2e-10,4.7] 0.12 0.12 0.12 0.12 0.12 0.12 0.12 0.12 0.032

288 G 0.2 [6.2e-10,4.7] 0.12 0.12 0.12 0.12 0.12 0.12 0.12 0.12 0.034

289 L 0.27 [6.2e-10,4.7] 0.12 0.12 0.12 0.12 0.12 0.12 0.12 0.12 0.049

290 L 0.23 [6.2e-10,4.7] 0.12 0.12 0.12 0.12 0.12 0.12 0.12 0.12 0.04

291 L 0.23 [6.2e-10,4.7] 0.12 0.12 0.12 0.12 0.12 0.12 0.12 0.12 0.04

292 H 0.27 [6.2e-10,4.7] 0.12 0.12 0.12 0.12 0.12 0.12 0.12 0.12 0.049

293 I 0.28 [6.2e-10,4.7] 0.12 0.12 0.12 0.12 0.12 0.12 0.12 0.12 0.05

294 H 0.27 [6.2e-10,4.7] 0.12 0.12 0.12 0.12 0.12 0.12 0.12 0.12 0.049

295 R 0.29 [6.2e-10,4.7] 0.12 0.12 0.12 0.12 0.12 0.12 0.12 0.12 0.054

296 A 0.24 [6.2e-10,4.7] 0.12 0.12 0.12 0.12 0.12 0.12 0.12 0.12 0.042

297 M 0.24 [6.2e-10,4.7] 0.12 0.12 0.12 0.12 0.12 0.12 0.12 0.12 0.043

298 H 0.21 [6.2e-10,4.7] 0.12 0.12 0.12 0.12 0.12 0.12 0.12 0.12 0.035

299 A 0.28 [6.2e-10,4.7] 0.12 0.12 0.12 0.12 0.12 0.12 0.12 0.12 0.051

300 V 0.2 [6.2e-10,4.7] 0.12 0.12 0.12 0.12 0.12 0.12 0.12 0.12 0.034

301 I 0.21 [6.2e-10,4.7] 0.12 0.12 0.12 0.12 0.12 0.12 0.12 0.12 0.036

302 D 0.17 [6.2e-10,4.7] 0.12 0.12 0.12 0.12 0.12 0.12 0.12 0.12 0.029

303 R 0.25 [6.2e-10,4.7] 0.12 0.12 0.12 0.12 0.12 0.12 0.12 0.12 0.044

304 Q 0.27 [6.2e-10,4.7] 0.12 0.12 0.12 0.12 0.12 0.12 0.12 0.12 0.049

305 K 0.26 [6.2e-10,4.7] 0.12 0.12 0.12 0.12 0.12 0.12 0.12 0.12 0.046

306 N 0.19 [6.2e-10,4.7] 0.12 0.12 0.12 0.12 0.12 0.12 0.12 0.12 0.032

307 H 0.21 [6.2e-10,4.7] 0.12 0.12 0.12 0.12 0.12 0.12 0.12 0.12 0.035

308 G 0.2 [6.2e-10,4.7] 0.12 0.12 0.12 0.12 0.12 0.12 0.12 0.12 0.034

309 M 0.24 [6.2e-10,4.7] 0.12 0.12 0.12 0.12 0.12 0.12 0.12 0.12 0.043

310 H 0.27 [6.2e-10,4.7] 0.12 0.12 0.12 0.12 0.12 0.12 0.12 0.12 0.049

311 F 0.28 [6.2e-10,4.7] 0.12 0.12 0.12 0.12 0.12 0.12 0.12 0.12 0.05

312 R 0.23 [6.2e-10,4.7] 0.12 0.12 0.12 0.12 0.12 0.12 0.12 0.12 0.041

313 V 0.24 [6.2e-10,4.7] 0.12 0.12 0.12 0.12 0.12 0.12 0.12 0.12 0.042

314 L 0.27 [6.2e-10,4.7] 0.12 0.12 0.12 0.12 0.12 0.12 0.12 0.12 0.049

315 A 0.2 [6.2e-10,4.7] 0.12 0.12 0.12 0.12 0.12 0.12 0.12 0.12 0.034

316 K 0.23 [6.2e-10,4.7] 0.12 0.12 0.12 0.12 0.12 0.12 0.12 0.12 0.039

317 A 0.28 [6.2e-10,4.7] 0.12 0.12 0.12 0.12 0.12 0.12 0.12 0.12 0.051

318 L 0.27 [6.2e-10,4.7] 0.12 0.12 0.12 0.12 0.12 0.12 0.12 0.12 0.049

319 R 0.23 [6.2e-10,4.7] 0.12 0.12 0.12 0.12 0.12 0.12 0.12 0.12 0.041

320 M 0.24 [6.2e-10,4.7] 0.12 0.12 0.12 0.12 0.12 0.12 0.12 0.12 0.043

321 S 0.29 [6.2e-10,4.7] 0.12 0.12 0.12 0.12 0.12 0.12 0.12 0.12 0.054

322 G 0.2 [6.2e-10,4.7] 0.12 0.12 0.12 0.12 0.12 0.12 0.12 0.12 0.034

323 G 0.24 [6.2e-10,4.7] 0.12 0.12 0.12 0.12 0.12 0.12 0.12 0.12 0.043

324 D 0.17 [6.2e-10,4.7] 0.12 0.12 0.12 0.12 0.12 0.12 0.12 0.12 0.029

325 H 0.27 [6.2e-10,4.7] 0.12 0.12 0.12 0.12 0.12 0.12 0.12 0.12 0.049

326 I 0.21 [6.2e-10,4.7] 0.12 0.12 0.12 0.12 0.12 0.12 0.12 0.12 0.036

327 H 0.21 [6.2e-10,4.7] 0.12 0.12 0.12 0.12 0.12 0.12 0.12 0.12 0.035

328 A 4.1 [0.054,4.7] 2.6e-10 1.9e-07 8.6e-06 0.00013 0.00099 0.0053 0.023 0.11 0.86

329 G 0.2 [6.2e-10,4.7] 0.12 0.12 0.12 0.12 0.12 0.12 0.12 0.12 0.034

330 T 0.28 [6.2e-10,4.7] 0.12 0.12 0.12 0.12 0.12 0.12 0.12 0.12 0.052

331 V 0.24 [6.2e-10,4.7] 0.12 0.12 0.12 0.12 0.12 0.12 0.12 0.12 0.042

332 V 0.24 [6.2e-10,4.7] 0.12 0.12 0.12 0.12 0.12 0.12 0.12 0.12 0.042

333 G 0.2 [6.2e-10,4.7] 0.12 0.12 0.12 0.12 0.12 0.12 0.12 0.12 0.034

334 K 0.23 [6.2e-10,4.7] 0.12 0.12 0.12 0.12 0.12 0.12 0.12 0.12 0.039

335 L 0.23 [6.2e-10,4.7] 0.12 0.12 0.12 0.12 0.12 0.12 0.12 0.12 0.04

336 E 0.21 [6.2e-10,4.7] 0.12 0.12 0.12 0.12 0.12 0.12 0.12 0.12 0.036

337 G 0.28 [6.2e-10,4.7] 0.12 0.12 0.12 0.12 0.12 0.12 0.12 0.12 0.051

338 E 0.21 [6.2e-10,4.7] 0.12 0.12 0.12 0.12 0.12 0.12 0.12 0.12 0.036

339 R 0.25 [6.2e-10,4.7] 0.12 0.12 0.12 0.12 0.12 0.12 0.12 0.12 0.044

340 E 0.21 [6.2e-10,4.7] 0.12 0.12 0.12 0.12 0.12 0.12 0.12 0.12 0.036

341 I 0.28 [6.2e-10,4.7] 0.12 0.12 0.12 0.12 0.12 0.12 0.12 0.12 0.05

342 T 0.22 [6.2e-10,4.7] 0.12 0.12 0.12 0.12 0.12 0.12 0.12 0.12 0.037

343 L 0.29 [6.2e-10,4.7] 0.12 0.12 0.12 0.12 0.12 0.12 0.12 0.12 0.053

344 G 0.28 [6.2e-10,4.7] 0.12 0.12 0.12 0.12 0.12 0.12 0.12 0.12 0.05

345 F 0.22 [6.2e-10,4.7] 0.12 0.12 0.12 0.12 0.12 0.12 0.12 0.12 0.038

346 V 0.2 [6.2e-10,4.7] 0.12 0.12 0.12 0.12 0.12 0.12 0.12 0.12 0.034

347 D 0.17 [6.2e-10,4.7] 0.12 0.12 0.12 0.12 0.12 0.12 0.12 0.12 0.029

348 L 0.27 [6.2e-10,4.7] 0.12 0.12 0.12 0.12 0.12 0.12 0.12 0.12 0.049

349 L 0.3 [6.2e-10,4.7] 0.12 0.12 0.12 0.12 0.12 0.12 0.12 0.12 0.055

350 R 0.23 [6.2e-10,4.7] 0.12 0.12 0.12 0.12 0.12 0.12 0.12 0.12 0.041

351 D 0.17 [6.2e-10,4.7] 0.12 0.12 0.12 0.12 0.12 0.12 0.12 0.12 0.029

352 D 0.17 [6.2e-10,4.7] 0.12 0.12 0.12 0.12 0.12 0.12 0.12 0.12 0.029

353 F 0.22 [6.2e-10,4.7] 0.12 0.12 0.12 0.12 0.12 0.12 0.12 0.12 0.038

354 I 0.21 [6.2e-10,4.7] 0.12 0.12 0.12 0.12 0.12 0.12 0.12 0.12 0.036

355 E 0.21 [6.2e-10,4.7] 0.12 0.12 0.12 0.12 0.12 0.12 0.12 0.12 0.036

356 K 0.23 [6.2e-10,4.7] 0.12 0.12 0.12 0.12 0.12 0.12 0.12 0.12 0.039

357 D 0.17 [6.2e-10,4.7] 0.12 0.12 0.12 0.12 0.12 0.12 0.12 0.12 0.029

358 R 0.25 [6.2e-10,4.7] 0.12 0.12 0.12 0.12 0.12 0.12 0.12 0.12 0.044

359 S 0.21 [6.2e-10,4.7] 0.12 0.12 0.12 0.12 0.12 0.12 0.12 0.12 0.035

360 R 0.29 [6.2e-10,4.7] 0.12 0.12 0.12 0.12 0.12 0.12 0.12 0.12 0.054

361 G 0.2 [6.2e-10,4.7] 0.12 0.12 0.12 0.12 0.12 0.12 0.12 0.12 0.034

362 I 0.21 [6.2e-10,4.7] 0.12 0.12 0.12 0.12 0.12 0.12 0.12 0.12 0.036

363 Y 0.22 [6.2e-10,4.7] 0.12 0.12 0.12 0.12 0.12 0.12 0.12 0.12 0.038

364 F 0.26 [6.2e-10,4.7] 0.12 0.12 0.12 0.12 0.12 0.12 0.12 0.12 0.046

365 T 0.28 [6.2e-10,4.7] 0.12 0.12 0.12 0.12 0.12 0.12 0.12 0.12 0.052

366 Q 0.25 [6.2e-10,4.7] 0.12 0.12 0.12 0.12 0.12 0.12 0.12 0.12 0.044

367 D 0.17 [6.2e-10,4.7] 0.12 0.12 0.12 0.12 0.12 0.12 0.12 0.12 0.029

368 W 0.29 [6.2e-10,4.7] 0.12 0.12 0.12 0.12 0.12 0.12 0.12 0.12 0.053

369 V 0.28 [6.2e-10,4.7] 0.12 0.12 0.12 0.12 0.12 0.12 0.12 0.12 0.05

370 S 0.23 [6.2e-10,4.7] 0.12 0.12 0.12 0.12 0.12 0.12 0.12 0.12 0.041

371 L 0.27 [6.2e-10,4.7] 0.12 0.12 0.12 0.12 0.12 0.12 0.12 0.12 0.049

372 P 0.26 [6.2e-10,4.7] 0.12 0.12 0.12 0.12 0.12 0.12 0.12 0.12 0.047

373 G 0.2 [6.2e-10,4.7] 0.12 0.12 0.12 0.12 0.12 0.12 0.12 0.12 0.034

374 V 0.22 [6.2e-10,4.7] 0.12 0.12 0.12 0.12 0.12 0.12 0.12 0.12 0.037

375 I 4.5 [0.26,4.7] 6.8e-11 6.4e-08 2.9e-06 4.3e-05 0.00033 0.0018 0.0079 0.042 0.95

376 P 0.23 [6.2e-10,4.7] 0.12 0.12 0.12 0.12 0.12 0.12 0.12 0.12 0.041

377 V 0.24 [6.2e-10,4.7] 0.12 0.12 0.12 0.12 0.12 0.12 0.12 0.12 0.042

378 A 0.2 [6.2e-10,4.7] 0.12 0.12 0.12 0.12 0.12 0.12 0.12 0.12 0.034

379 S 0.3 [6.2e-10,4.7] 0.12 0.12 0.12 0.12 0.12 0.12 0.12 0.12 0.055

380 G 0.28 [6.2e-10,4.7] 0.12 0.12 0.12 0.12 0.12 0.12 0.12 0.12 0.051

381 G 0.2 [6.2e-10,4.7] 0.12 0.12 0.12 0.12 0.12 0.12 0.12 0.12 0.034

382 I 0.21 [6.2e-10,4.7] 0.12 0.12 0.12 0.12 0.12 0.12 0.12 0.12 0.036

383 H 0.27 [6.2e-10,4.7] 0.12 0.12 0.12 0.12 0.12 0.12 0.12 0.12 0.049

384 V 0.2 [6.2e-10,4.7] 0.12 0.12 0.12 0.12 0.12 0.12 0.12 0.12 0.034

385 W 0.29 [6.2e-10,4.7] 0.12 0.12 0.12 0.12 0.12 0.12 0.12 0.12 0.053

386 H 0.21 [6.2e-10,4.7] 0.12 0.12 0.12 0.12 0.12 0.12 0.12 0.12 0.035

387 M 0.24 [6.2e-10,4.7] 0.12 0.12 0.12 0.12 0.12 0.12 0.12 0.12 0.043

388 P 0.23 [6.2e-10,4.7] 0.12 0.12 0.12 0.12 0.12 0.12 0.12 0.12 0.041

389 A 0.2 [6.2e-10,4.7] 0.12 0.12 0.12 0.12 0.12 0.12 0.12 0.12 0.034

390 L 0.27 [6.2e-10,4.7] 0.12 0.12 0.12 0.12 0.12 0.12 0.12 0.12 0.049

391 T 0.28 [6.2e-10,4.7] 0.12 0.12 0.12 0.12 0.12 0.12 0.12 0.12 0.052

392 E 0.24 [6.2e-10,4.7] 0.12 0.12 0.12 0.12 0.12 0.12 0.12 0.12 0.042

393 I 0.28 [6.2e-10,4.7] 0.12 0.12 0.12 0.12 0.12 0.12 0.12 0.12 0.05

394 F 0.22 [6.2e-10,4.7] 0.12 0.12 0.12 0.12 0.12 0.12 0.12 0.12 0.038

395 G 0.25 [6.2e-10,4.7] 0.12 0.12 0.12 0.12 0.12 0.12 0.12 0.12 0.045

396 D 0.17 [6.2e-10,4.7] 0.12 0.12 0.12 0.12 0.12 0.12 0.12 0.12 0.029

397 D 0.17 [6.2e-10,4.7] 0.12 0.12 0.12 0.12 0.12 0.12 0.12 0.12 0.029

398 S 0.29 [6.2e-10,4.7] 0.12 0.12 0.12 0.12 0.12 0.12 0.12 0.12 0.054

399 V 0.24 [6.2e-10,4.7] 0.12 0.12 0.12 0.12 0.12 0.12 0.12 0.12 0.042

400 L 0.27 [6.2e-10,4.7] 0.12 0.12 0.12 0.12 0.12 0.12 0.12 0.12 0.049

401 Q 0.27 [6.2e-10,4.7] 0.12 0.12 0.12 0.12 0.12 0.12 0.12 0.12 0.049

402 F 0.28 [6.2e-10,4.7] 0.12 0.12 0.12 0.12 0.12 0.12 0.12 0.12 0.05

403 G 0.2 [6.2e-10,4.7] 0.12 0.12 0.12 0.12 0.12 0.12 0.12 0.12 0.034

404 G 0.24 [6.2e-10,4.7] 0.12 0.12 0.12 0.12 0.12 0.12 0.12 0.12 0.043

405 G 0.24 [6.2e-10,4.7] 0.12 0.12 0.12 0.12 0.12 0.12 0.12 0.12 0.043

406 T 0.22 [6.2e-10,4.7] 0.12 0.12 0.12 0.12 0.12 0.12 0.12 0.12 0.037

407 L 0.27 [6.2e-10,4.7] 0.12 0.12 0.12 0.12 0.12 0.12 0.12 0.12 0.049

408 G 0.28 [6.2e-10,4.7] 0.12 0.12 0.12 0.12 0.12 0.12 0.12 0.12 0.051

409 H 0.27 [6.2e-10,4.7] 0.12 0.12 0.12 0.12 0.12 0.12 0.12 0.12 0.049

410 P 0.23 [6.2e-10,4.7] 0.12 0.12 0.12 0.12 0.12 0.12 0.12 0.12 0.041

411 W 0.29 [6.2e-10,4.7] 0.12 0.12 0.12 0.12 0.12 0.12 0.12 0.12 0.053

412 G 0.2 [6.2e-10,4.7] 0.12 0.12 0.12 0.12 0.12 0.12 0.12 0.12 0.034

413 N 0.19 [6.2e-10,4.7] 0.12 0.12 0.12 0.12 0.12 0.12 0.12 0.12 0.032

414 A 0.24 [6.2e-10,4.7] 0.12 0.12 0.12 0.12 0.12 0.12 0.12 0.12 0.042

415 P 0.23 [6.2e-10,4.7] 0.12 0.12 0.12 0.12 0.12 0.12 0.12 0.12 0.041

416 G 0.2 [6.2e-10,4.7] 0.12 0.12 0.12 0.12 0.12 0.12 0.12 0.12 0.034

417 A 0.28 [6.2e-10,4.7] 0.12 0.12 0.12 0.12 0.12 0.12 0.12 0.12 0.05

418 V 0.24 [6.2e-10,4.7] 0.12 0.12 0.12 0.12 0.12 0.12 0.12 0.12 0.042

419 A 0.2 [6.2e-10,4.7] 0.12 0.12 0.12 0.12 0.12 0.12 0.12 0.12 0.034

420 N 0.26 [6.2e-10,4.7] 0.12 0.12 0.12 0.12 0.12 0.12 0.12 0.12 0.046

421 R 0.28 [6.2e-10,4.7] 0.12 0.12 0.12 0.12 0.12 0.12 0.12 0.12 0.052

422 V 0.24 [6.2e-10,4.7] 0.12 0.12 0.12 0.12 0.12 0.12 0.12 0.12 0.042

423 A 0.2 [6.2e-10,4.7] 0.12 0.12 0.12 0.12 0.12 0.12 0.12 0.12 0.034

424 L 0.27 [6.2e-10,4.7] 0.12 0.12 0.12 0.12 0.12 0.12 0.12 0.12 0.049

425 E 0.21 [6.2e-10,4.7] 0.12 0.12 0.12 0.12 0.12 0.12 0.12 0.12 0.036

426 A 0.24 [6.2e-10,4.7] 0.12 0.12 0.12 0.12 0.12 0.12 0.12 0.12 0.042

427 C 0.24 [6.2e-10,4.7] 0.12 0.12 0.12 0.12 0.12 0.12 0.12 0.12 0.043

428 V 0.24 [6.2e-10,4.7] 0.12 0.12 0.12 0.12 0.12 0.12 0.12 0.12 0.042

429 Q 4 [0.054,4.7] 3e-10 2.2e-07 9.9e-06 0.00014 0.0011 0.0061 0.026 0.13 0.84

430 A 0.2 [6.2e-10,4.7] 0.12 0.12 0.12 0.12 0.12 0.12 0.12 0.12 0.034

431 R 0.23 [6.2e-10,4.7] 0.12 0.12 0.12 0.12 0.12 0.12 0.12 0.12 0.041

432 N 0.19 [6.2e-10,4.7] 0.12 0.12 0.12 0.12 0.12 0.12 0.12 0.12 0.032

433 E 0.24 [6.2e-10,4.7] 0.12 0.12 0.12 0.12 0.12 0.12 0.12 0.12 0.042

434 G 0.24 [6.2e-10,4.7] 0.12 0.12 0.12 0.12 0.12 0.12 0.12 0.12 0.043

435 R 0.29 [6.2e-10,4.7] 0.12 0.12 0.12 0.12 0.12 0.12 0.12 0.12 0.054

436 D 0.17 [6.2e-10,4.7] 0.12 0.12 0.12 0.12 0.12 0.12 0.12 0.12 0.029

437 L 0.23 [6.2e-10,4.7] 0.12 0.12 0.12 0.12 0.12 0.12 0.12 0.12 0.04

438 A 0.2 [6.2e-10,4.7] 0.12 0.12 0.12 0.12 0.12 0.12 0.12 0.12 0.034

439 T 0.22 [6.2e-10,4.7] 0.12 0.12 0.12 0.12 0.12 0.12 0.12 0.12 0.037

440 E 0.24 [6.2e-10,4.7] 0.12 0.12 0.12 0.12 0.12 0.12 0.12 0.12 0.042

441 G 0.2 [6.2e-10,4.7] 0.12 0.12 0.12 0.12 0.12 0.12 0.12 0.12 0.034

442 N 0.19 [6.2e-10,4.7] 0.12 0.12 0.12 0.12 0.12 0.12 0.12 0.12 0.032

443 E 0.21 [6.2e-10,4.7] 0.12 0.12 0.12 0.12 0.12 0.12 0.12 0.12 0.036

444 I 0.21 [6.2e-10,4.7] 0.12 0.12 0.12 0.12 0.12 0.12 0.12 0.12 0.036

445 I 0.28 [6.2e-10,4.7] 0.12 0.12 0.12 0.12 0.12 0.12 0.12 0.12 0.05

446 R 0.28 [6.2e-10,4.7] 0.12 0.12 0.12 0.12 0.12 0.12 0.12 0.12 0.051

447 E 0.24 [6.2e-10,4.7] 0.12 0.12 0.12 0.12 0.12 0.12 0.12 0.12 0.042

448 A 0.2 [6.2e-10,4.7] 0.12 0.12 0.12 0.12 0.12 0.12 0.12 0.12 0.034

449 T 4.1 [0.054,4.7] 2.6e-10 1.9e-07 8.5e-06 0.00012 0.00097 0.0052 0.023 0.11 0.86

450 K 0.23 [6.2e-10,4.7] 0.12 0.12 0.12 0.12 0.12 0.12 0.12 0.12 0.039

451 W 0.29 [6.2e-10,4.7] 0.12 0.12 0.12 0.12 0.12 0.12 0.12 0.12 0.053

452 S 0.21 [6.2e-10,4.7] 0.12 0.12 0.12 0.12 0.12 0.12 0.12 0.12 0.035

453 P 0.23 [6.2e-10,4.7] 0.12 0.12 0.12 0.12 0.12 0.12 0.12 0.12 0.041

454 E 0.21 [6.2e-10,4.7] 0.12 0.12 0.12 0.12 0.12 0.12 0.12 0.12 0.036

455 L 0.27 [6.2e-10,4.7] 0.12 0.12 0.12 0.12 0.12 0.12 0.12 0.12 0.049

456 A 0.2 [6.2e-10,4.7] 0.12 0.12 0.12 0.12 0.12 0.12 0.12 0.12 0.034

457 A 0.2 [6.2e-10,4.7] 0.12 0.12 0.12 0.12 0.12 0.12 0.12 0.12 0.034

458 A 0.2 [6.2e-10,4.7] 0.12 0.12 0.12 0.12 0.12 0.12 0.12 0.12 0.034

459 C 0.23 [6.2e-10,4.7] 0.12 0.12 0.12 0.12 0.12 0.12 0.12 0.12 0.041

460 E 0.21 [6.2e-10,4.7] 0.12 0.12 0.12 0.12 0.12 0.12 0.12 0.12 0.036

461 V 0.24 [6.2e-10,4.7] 0.12 0.12 0.12 0.12 0.12 0.12 0.12 0.12 0.042

462 W 0.29 [6.2e-10,4.7] 0.12 0.12 0.12 0.12 0.12 0.12 0.12 0.12 0.053

463 K 0.26 [6.2e-10,4.7] 0.12 0.12 0.12 0.12 0.12 0.12 0.12 0.12 0.046

464 E 0.24 [6.2e-10,4.7] 0.12 0.12 0.12 0.12 0.12 0.12 0.12 0.12 0.042

465 I 0.28 [6.2e-10,4.7] 0.12 0.12 0.12 0.12 0.12 0.12 0.12 0.12 0.05

466 K 0.23 [6.2e-10,4.7] 0.12 0.12 0.12 0.12 0.12 0.12 0.12 0.12 0.039

467 F 0.22 [6.2e-10,4.7] 0.12 0.12 0.12 0.12 0.12 0.12 0.12 0.12 0.038

468 E 0.23 [6.2e-10,4.7] 0.12 0.12 0.12 0.12 0.12 0.12 0.12 0.12 0.041

469 F 0.28 [6.2e-10,4.7] 0.12 0.12 0.12 0.12 0.12 0.12 0.12 0.12 0.05

470 Q 0.27 [6.2e-10,4.7] 0.12 0.12 0.12 0.12 0.12 0.12 0.12 0.12 0.049

471 A 0.24 [6.2e-10,4.7] 0.12 0.12 0.12 0.12 0.12 0.12 0.12 0.12 0.042

472 M 0.24 [6.2e-10,4.7] 0.12 0.12 0.12 0.12 0.12 0.12 0.12 0.12 0.043

473 D 0.17 [6.2e-10,4.7] 0.12 0.12 0.12 0.12 0.12 0.12 0.12 0.12 0.029

474 T 0.22 [6.2e-10,4.7] 0.12 0.12 0.12 0.12 0.12 0.12 0.12 0.12 0.037

475 L 0.29 [6.2e-10,4.7] 0.12 0.12 0.12 0.12 0.12 0.12 0.12 0.12 0.053

476 D 0.17 [6.2e-10,4.7] 0.12 0.12 0.12 0.12 0.12 0.12 0.12 0.12 0.029

477 G 0.28 [6.2e-10,4.7] 0.12 0.12 0.12 0.12 0.12 0.12 0.12 0.12 0.051

478 D 0.17 [6.2e-10,4.7] 0.12 0.12 0.12 0.12 0.12 0.12 0.12 0.12 0.029

479 K 0.26 [6.2e-10,4.7] 0.12 0.12 0.12 0.12 0.12 0.12 0.12 0.12 0.046

480 D 0.17 [6.2e-10,4.7] 0.12 0.12 0.12 0.12 0.12 0.12 0.12 0.12 0.029

481 K 0.23 [6.2e-10,4.7] 0.12 0.12 0.12 0.12 0.12 0.12 0.12 0.12 0.039

482 D 0.17 [6.2e-10,4.7] 0.12 0.12 0.12 0.12 0.12 0.12 0.12 0.12 0.029

483 K 0.26 [6.2e-10,4.7] 0.12 0.12 0.12 0.12 0.12 0.12 0.12 0.12 0.046

484 K 0.26 [6.2e-10,4.7] 0.12 0.12 0.12 0.12 0.12 0.12 0.12 0.12 0.046

485 R 0.25 [6.2e-10,4.7] 0.12 0.12 0.12 0.12 0.12 0.12 0.12 0.12 0.044
